# Low physical activity-related disease burden, 1990–2021: assessment of global trends and social determinants based on GBD 2021 data

**DOI:** 10.7189/jogh.15.04314

**Published:** 2025-12-05

**Authors:** Hao Liu, Zhenhao Liu, Yanqing Gong, Jingbin Guo, Xin Liu, Yu Sun, Weiming Tang, Weibin Cheng, Wen Jin

**Affiliations:** 1 The Affiliated Guangdong Second Provincial General Hospital of Jinan University, Guangzhou, Guangdong, China; 2 Department of Cardiovascular Medicine, Pingxiang People's Hospital, Jiangxi, China; 3 The Second Clinical Medical School, Southern Medical University, Guangzhou, China; 4 Department of Cardiology, Laboratory of Heart Center, Zhujiang Hospital, Southern Medical University, Guangzhou, China; 5 Department of Medical Engineering, Zhujiang Hospital, Southern Medical University, Guangzhou, China; 6 Department of Cardiac Intensive Care Unit, the Cardiovascular Hospital, the Affiliated Guangdong Second Provincial General Hospital of Jinan University, Guangzhou, Guangdong, China; 7 Institute of Global Health and Infectious Diseases, University of North Carolina at ChapelHill, Chapel Hill, North Carolina, USA; 8 Institute for Healthcare Artificial Intelligence Application, The Affiliated Guangdong Second Provincial General Hospital of Jinan University, Guangzhou, Guangdong, China

## Abstract

**Background:**

Low physical activity (LPA) is associated with cardiovascular and cerebrovascular pathologies. This study aimed to assess the prevalence of several noncommunicable diseases relating to LPA.

**Methods:**

Using the 2021 Global Burden of Disease data set, we modelled LPA-related disease burdens across 204 countries and territories, quantifying mortality counts, age-standardised mortality rates, and disability-adjusted life years (DALYs) for five noncommunicable diseases. We conducted multivariable stratification analyses to assess variations by gender, age, and sociodemographic index (SDI) quintiles. We used age-period-cohort modelling to project burden trajectories, while applying counterfactual decomposition frameworks to delineate synergistic interactions between LPA and risk factors.

**Results:**

We found that LPA accounted for 555 101 related deaths globally in 2021 across the five studied pathologies, mostly among individuals aged 60–94 years. Association between LPA-related disease burden and SDI followed a U-shaped distribution across regions and diseases. Among individuals aged 60–89 years, LPA-related deaths were significantly higher in women than in men, indicating a disproportionate burden on elderly females. Ischaemic heart disease (IHD) trends stabilised in low- and middle-SDI regions but declined significantly in high-SDI regions, underscoring global health disparities. From 2007 to 2011, LPA DALYs and mortality risk ratios for IHD, stroke, and lower extremity peripheral arterial disease declined from >1 to <1, whereas diabetes mellitus exhibited an opposite trend, highlighting LPA’s persistent and significant impact on diabetes-related morbidity. Demographic shifts and epidemiological transitions were primary drivers of LPA-related disease burden across five pathologies. In high-SDI regions, epidemiological changes predominated, whereas population growth was a key factor in low- and middle-SDI regions. Synergistic interaction of these factors with LPA is projected to substantially amplify future disease burden.

**Conclusions:**

Physical activity should be increased among elderly women to address health risks associated with LPA. Likewise, urgent public health interventions are needed for LPA-related diabetes. As IHD burden rises in low- and middle-SDI regions, vascular disease care strategies require optimisation. Moreover, high-SDI regions should strengthen nationwide physical activity promotion, while low- and middle-SDI areas must enhance healthcare infrastructure and manage population growth to reduce LPA-related disease burdens.

Ischemic heart disease (IHD), stroke, lower extremity peripheral arterial disease (LEPAD), diabetes mellitus, and chronic kidney disease (CKD) have become the main causes of death and disability globally [1,2]. According to research conducted by Jie Li and colleagues in 2021, IHD caused approximately 9.0 million deaths. Stroke accounted for ~ 7.3 million deaths, diabetes mellitus led to roughly 1.7 million fatalities, CKD resulted in ~ 1.5 million deaths, and LEPAD caused ~ 0.1 million deaths [1]. Moreover, the number of people suffering from disabilities due to these diseases reached several million or even more [1]. The development of these diseases is influenced by multiple factors, encompassing age, genetic predispositions, environmental exposures, and lifestyle choices [3]. According to the 2023 American Heart Association report, age is a critical risk factor for IHD and stroke, with older patients generally experiencing poorer prognoses [4]. Furthermore, the incidence of diabetes and CKD is closely linked to age [5]. For instance, among individuals aged 75 years and older, the incidence of CKD exceeds 3000 cases per 100 000 people [6]. The prevalence of diabetes mellitus also increases with age, and the longer the disease duration, the higher the risk of adverse cardiovascular events [7,8].

Physical activity is any bodily movement generated by skeletal muscles that leads to energy consumption [9]. It comes in diverse forms, such as daily activities, work-related physical labour, recreational pursuits, and structured exercises [9]. According to the World Health Organization (WHO) 2020 guidelines, adults are advised to perform at least 150–300 minutes of moderate-intensity or 75–150 minutes of vigorous-intensity aerobic physical activity, or an equivalent mix of both intensities, at least three times a week [9]. Physical activity can enhance cardiovascular health and lower the risk of IHD [10]. For patients with diabetes mellitus, it aids in blood glucose control and improves the prognosis of diabetes-related complications [11]. Research shows that pre-stroke physical activity is linked to lower post-stroke mortality [12], with one long-term follow-up study indicating that higher levels of physical activity are associated with a reduced risk of CKD [13]. Moreover, Chinese scholars' research indicates that engaging in moderate to vigorous physical activity can significantly cut the risk of LEPAD [14]. However, for various reasons, many people do not meet these standards and are classified as having low physical activity (LPA) [15,16], which can elevate the risk of chronic diseases like cardiovascular diseases, diabetes mellitus, and stroke [9].

Physical inactivity significantly impacts the global burden of IHD [17]. As of 2019, LPA-related IHD accounted for 7.6 million disability-adjusted life years (DALYs) worldwide, mainly concentrated in countries with moderate and high socio-demographic index (SDI), with the lowest burden observed in low-SDI countries [17]. Moreover, LPA significantly affects the global burden of stroke [18]. In 2019, LPA-related stroke caused 152 000 deaths and 2 409 000 DALYs globally, with both being more notable among women and individuals over 70 years of age [18]. Furthermore, LPA impacts the diabetes mellitus burden by increasing DALYs worldwide, more so in low and middle-SDI regions than in high-SDI regions [19]. A study found that LPA is closely linked to the incidence and mortality of LEPAD, especially in low- and middle-income countries and among the elderly [20]. Finally, the data from the Global Burden of Disease (GBD) 2019 indicate that CKD exacerbated by LPA has led to a significant increase in both deaths and DALYs over the past few decades, especially among the elderly population [21].

While LPA accounts for the burden of five key diseases, emerging global patterns of LPA-related disease burden and their interactions with modifiable social determinants remain poorly characterised, hampering both clinical understanding and health system preparedness. Leveraging WHO/World Bank GBD 2021 data encompassing mortality and DALYs for five LPA-associated conditions (IHD, diabetes mellitus, stroke, LEPAD, CKD), we quantified their global-to-national burdens through age-standardised death rates (ASDRs) and age-standardised mortality rates (ASMRs), and estimated annual percentage changes (EAPCs) during 1990–2021, while interrogating demographic patterns (age/gender/SDI stratification), temporal trends via age-period-cohort modelling, and modifiable social determinants through decomposition analysis. We aimed to systematically clarify the impact of LPA on these diseases across five non-communicable diseases (including LEPAD and SKD) and provide the latest global data related to the SDI.

## METHODS

As a reanalysis of secondary data, our study adhered to JoGH’s Guidelines for Reporting Analyses of Big Data Repositories Open to Public (GRABDROP) (Table S1 in the **Online Supplementary Document**).

### Data sources

We utilised data from the GBD 2021 database [15], a leading global resource for disease burden evaluation, which covers 369 diseases and injuries across 204 countries from 1990 to 2021 and assesses 88 risk factors, while offering comprehensive data on incidence, prevalence, mortality, and DALYs.

### Definition of low physical activity

Physical activity is quantified by total weekly metabolic equivalent (MET) minutes, calculated by adding the frequency, duration of each activity, and METs corresponding to its intensity [16]. One MET equals the energy cost of quiet sitting, at 1 kcal/kg/h [16]. The recognised threshold for physical inactivity is <600 MET min/week; however, this may not account for all increased mortality risks associated with insufficient physical activity [22]. In the 2021 GBD study, physical activity data were analysed using the best published and unpublished epidemiological evidence on relative risks, along with the lowest physical activity levels observed in cohorts, to determine a single exposure level that minimises all-cause mortality risk. This process established the theoretical minimum risk exposure level (TMREL) [15], estimated at 600 MET min/week, which corresponds to the period with the fewest deaths across various outcomes [15,16]. Consequently, in the GBD study, LPA is defined as below TMREL.

### Case definition

IHD restricts the heart's blood supply, often due to coronary artery narrowing from atherosclerosis, impeding blood flow. The GBD study defines IHD as the aggregate of discrete sequelae, such as myocardial infarction, angina pectoris, or ischaemic cardiomyopathy [23]. For GBD 2021, we modelled the deaths and DALYs of acute myocardial infarction and chronic IHD. In GBD 2021, LEPAD was defined by an ankle-brachial index (ABI)≤0.9. Clinically, intermittent claudication was defined as exertional leg pain in individuals with an ABI below this threshold [23]. In line with WHO criteria, stroke is defined as the rapid onset of focal (or rarely global) cerebral function disturbances with clinical signs lasting over 24 hours, causing death, and with vascular origin as the sole apparent cause. Transient ischaemic attack cases are excluded [23]. CKD is defined by permanent loss of kidney function, measured using estimated glomerular filtration rate (eGFR) and urinary albumin-to-creatinine ratio. The CKD-EPI eGFR equation is considered the gold standard for individuals aged 18 years and older, while the Schwartz equation is used for those aged under 18 years [23]. The GBD 2021 study includes six CKD stages based on the degree of kidney function loss or the use of kidney replacement therapy [23]. According to the study, diabetes mellitus encompasses type 1 and type 2 diabetes, and with LPA selected as the exposure factor, only type 2 diabetes data remain in the database. Thus, in this context specifically, diabetes mellitus denotes type 2 diabetes, defined by GBD 2021 as a metabolic disorder where the body's insulin response is abnormal, resulting in chronic hyperglycemia that can cause long-term damage to the heart, blood vessels, eyes, kidneys, and nerves [23]. Finally, for this study, we selected participants aged 45 years and older, following the WHO’s definition in the Study on Global Ageing and Adult Health, in which this age group, *i.e.* the middle-aged and elderly, allows for a better analysis of age-related health problems [24].

### SDI definition

The SDI quantifies regional development on an annual scale, spanning from 0 (less developed) to 1 (developed) [21]. Derived from lagged *per capita* income, education for individuals aged 15 and older, and fertility rates among those under the age of 25, the measure categorises 204 countries/regions into five quintiles: low, low-middle, middle, high-middle, and high [21].

### Data extraction and disease model

We retrieved data on the burden of these five diseases (IHD, stroke, LEPAD, diabetes mellitus, CKD) among individuals aged 45 years and older from 1990 to 2021, considering LPA as an exposure, from the Global Health Data Exchange (GHDx) platform [15,25]. The data set includes mortality and DALYs by age, gender, and location, and GHDx sourced SDI data, based on income, education, and fertility, which classifies regions into five levels for socioeconomic health disparity analysis.

### Estimation of disease burden

We used the GBD Comparative Risk Assessment Framework to estimate deaths and DALYs linked to LPA [23]. This framework matches LPA with known disease-specific outcomes, requiring sufficient evidence of a causal link. Based on GBD 2021 pooled cohort studies, IHD, stroke, type 2 diabetes, CKD, and LEPAD are significantly associated with LPA [23]. DALYs quantify the overall health loss from LPA, calculated by adding years lived with disability and years of life lost [26], with one DALY equaling the loss of one year of full health [26]. DisMod-MR, version 2.1 (Institute for Health Metrics and Evaluation, Seattle, WA, USA), a Bayesian meta-regression tool originally designed for the GBD study, estimates deaths and DALYs attributable to LPA. The proportions of deaths and DALYs due to LPA are estimated via the population attributable fraction, indicating the reduction if LPA exposure reached the ideal scenario [23,26]. Details of the modelling strategy are available elsewhere [23].

Age-standardisation allows for comparing rates across locations or time. In GBD 2021, age-standardised rates were calculated via direct standardisation using the global age structure. Following Achraf Ammar’s approach, locally weighted scatterplot smoothing regression analysed the relationships between the SDI and age-standardised death proportions, as well as DALYs from LPA [21]. Uncertainty was evaluated through 1000 bootstrap samples at each calculation step [21]. The mean of these 1000 samples served as the estimate, with the 95% uncertainty interval (UI) defined by the 2.5th and 97.5th percentiles. Estimates with 95% UIs excluding zero were deemed statistically significant [21].

### Age-period-cohort (APC) analysis

The APC is a statistical model for analysing trends in disease mortality and DALYs rate over time, decomposing observed changes into age, period, and birth cohort factors [1].

From 1990 to 2021, the EAPCs were calculated via log-linear regression of the age-standardised rate (ASR). The EAPCs’ formula is (exp(b)−1) × 100, with a 95% confidence interval (CI) for the regression estimate. When the upper 95% confidence limit of EAPCs is below 0, there is a significant decline; when the lower limit exceeds 1, there is a significant increase [1].

### Decomposition analysis

Following the methodology of previous research [27], we used the Das Gupta decomposition analysis to understand the influencing factors that may interact with LPA]. This method quantifies the contributions of population ageing, growth, and epidemiological changes to the overall disease burden shift. By isolating variables, it evaluates each factor's independent impact, with the combined effects explaining the total burden change, thereby revealing their potential collaborative effects in relation to LPA [1].

Based on the GBD database, we included four multi-dimensional analyses: descriptive analyses (global/regional/national/SDI levels), sex-stratified analyses, APC analyses, and decomposition analyses (for independent factor effects). We applied the Bonferroni correction for multiple testing to control the family-wise error rate at ≤0.05. We calculated the corrected significance threshold (αₙ) as αₙ = 0.05/k(k = total number of independent tests, summed from comparisons/strata in each analysis). We used this threshold to avoid false positives in all inferential analyses (*e.g.* APC trend tests, decomposition factor significance tests).

## RESULTS

### Analysis of the overall burden of LPA globally and the burden of each LPA-related disease

**Figure 1:**
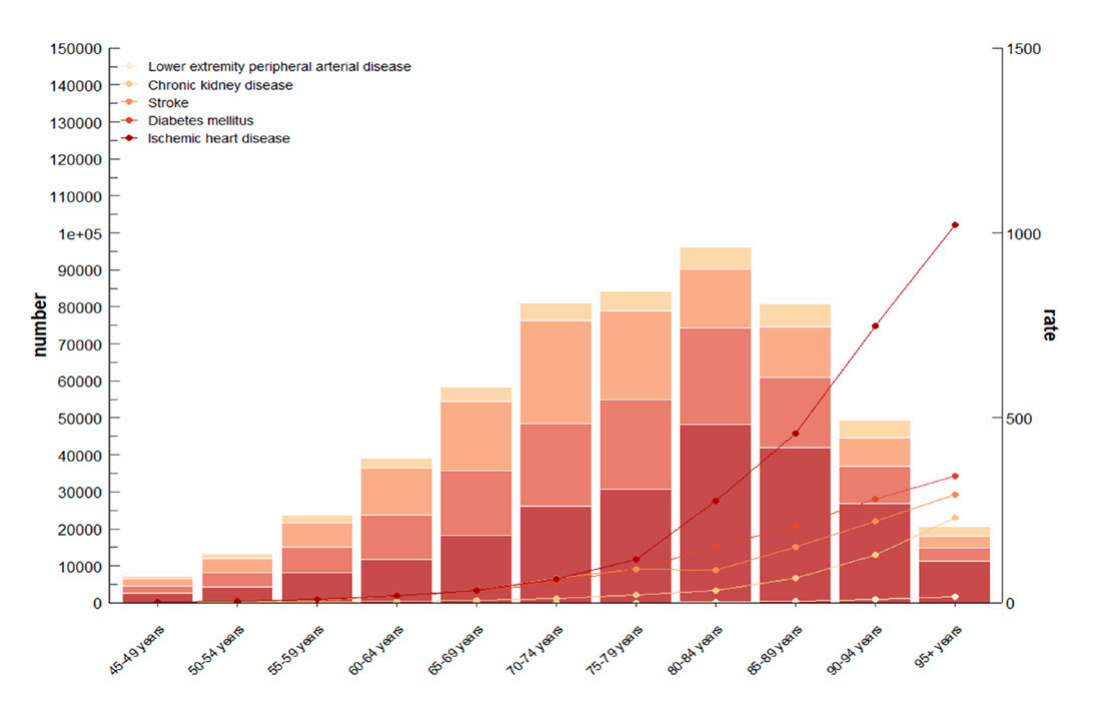
The distribution and trends of the number of deaths and mortality rates related to LPA in five diseases (LEPAD, CKD, stroke, diabetes mellitus, and IHD) among different age groups of the global population aged 45 years and above. CKD – chronic kidney disease, IHD – ischemic heart disease, LEPAD – lower extremity peripheral artery disease, LPA – low physical activity.

In 2021, the number of global deaths attributable to LPA rose with age, reaching its highest levels among those ~ 85 years. Most deaths occurred among individuals 60–94 years old. The total number of patients affected by LPA globally was ~ 555 101, a roughly 95% increase in the death proportion compared to 1990. In 2021, approximately 229 586 IHD deaths, 147 907 diabetes mellitus deaths, 135 766 stroke deaths, 40 145 chronic kidney disease deaths, and 1697 LEPAD deaths were related to LPA. Moreover, the LPA-related mortality rate in IHD increased exponentially, especially among patients over 80 (**Figure 1**). The LPA-related death burden of diabetes mellitus concentrated in individuals 75–89 years old, but improved among those 90 years old and older. Furthermore, the LPA-related mortality rate in stroke rose significantly in the 60–74 age group (**Figure 1**). After age standardisation in 2021, the LPA-related ASMRs for each disease were: 10.41 per 100 000 for IHD (95% UI = 4.54, 17.07), 5.9 for stroke (95% UI = −3.38, 15.17), 0.08 for LEPAD (95% UI = 0.02, 0.15), 6.52 for diabetes mellitus (95% UI = 2.8, 10.07), and 1.8 for CKD (95% UI = 0.67, 3.28) (**Table 1**). Globally, from 1990 to 2021, the EAPCs of LPA-related ASMRs decreased for IHD, stroke, and LEPAD (−1.19 (95% CI = −1.24, −1.15), −1.77 (95% CI = −1.88, −1.67), −2.78 (95% CI = −2.93, −2.63)) and increased for diabetes mellitus and CKD (0.25 (95% CI = 0.2, 0.31) and 0.64 (95% CI = 0.56, 0.72)) (**Table 1**).

**Table 1:** Global death and DALYs associated with LPA for five diseases (1990–2021)*

Year	Ischemic heart disease	Diabetes mellitus	Stroke	Chronic kidney disease	LEPAD
1990					
*Deaths*	123 863 (52 950, 201 328)	55 146 (23 698, 84 352)	90 346 (−31 089, 209 511)	13 435 (4879, 24740)	1291 (375, 2506)
*DALYs*	2 149 825 (932 677, 3 454 358)	1 664 981 (712 714, 2 595 137)	2 059 855 (35 568, 4 082 172)	330 374 (119 650, 611 690)	28 225 (7801, 56 466)
*ASMRs (95% UI)*	14.82 (6.27, 24.26)	5.94 (2.56, 9.09)	9.5 (−5.69, 24.28)	1.51 (0.55, 2.78)	0.17 (0.05, 0.33)
*ASDRs (95% UI)*	228.86 (98.4, 369.97)	161.99 (69.42, 252.53)	198.62 (−27.49, 422.35)	33.4 (12.15, 61.64)	3.15 (0.88, 6.27)
2021					
Death	229 586 (100 303, 376 343)	147 907 (63 453, 228 403)	135 766 (−65 733, 337 963)	40 145 (14974, 73094)	1697 (488, 3331)
*DALYs*	3 758 534 (1 668 371, 6 122 182)	523 8421 (2 280 033, 8 294 823)	3 172 145 (−148 487, 6 581 638)	856 532 (313 039, 1 565 898)	38 030 (10 501, 76 868)
*ASMRs (95% UI)*	10.41 (4.54,17.07)	6.52 (2.8,10.07)	5.9 (−3.38, 15.17)	1.8 (0.67, 3.28)	0.08 (0.02, 0.15)
*ASDRs (95% UI)*	164.37 (72.81,268.1)	222.4 (96.82, 351.97)	133.79 (−13.86, 284.8)	36.99 (13.55, 67.56)	1.68 (0.46, 3.39)
1990–2021					
*ASMRs (EAPCs, 95% CI)*	−1.19 (−1.24, −1.15)	0.25 (0.2, 0.31)	−1.77 (−1.88, −1.67)	0.64 (0.56, 0.72)	−2.78 (−2.93, −2.63)
*ASDRs (EAPCs, 95% CI)*	−1.16 (−1.22, −1.11)	0.92 (0.87, 0.96)	−1.52 (−1.61, −1.43)	0.37 (0.31, 0.44)	−2.38 (−2.49, −2.27)

ASDRs – age-standardised death rates, ASMRs– age-standardised mortality rates, CI – confidence interval, DALYs – disability-adjusted life years, EAPCs – estimated annual percentage changes, LEPAD – lower extremity peripheral artery disease, UI – uncertainty interval

*Values presented as n (95% UI) unless specified otherwise.

In addition, as of 2021, the LPA-related DALYs differed among the five diseases: 3.75 million for IHD, 3.17 million for stroke, 5.23 million for diabetes mellitus, 0.85 million for CKD, and 0.03 million for LEPAD (**Table 1**). After age standardisation, the LPA-related ASDRs for IHD, stroke, diabetes mellitus, CKD, and LEPAD were 164.37 (95% UI = 72.81, 268.1), 133.79 (95% UI = −13.86, 284.8), 222.4 (95% UI = 96.82, 351.97), 36.99 (95% UI = 13.55, 67.56), and 1.68 (95% UI = 0.46, 3.39), respectively (**Table 1**).

From 1990 to 2021, the EAPCs of the LPA-related ASDRs for these five diseases were −1.16 (95% CI = −1.22, −1.11), −1.52 (95% CI = −1.61, −1.43), 0.92 (95% CI = 0.87, 0.96), 0.37 (95% CI = 0.31, 0.44), and −2.38 (95% CI = −2.49, −2.27), respectively (**Table 1**).

### Analysis of the burden of each LPA-related disease within the regional scope

**Figure 2:**
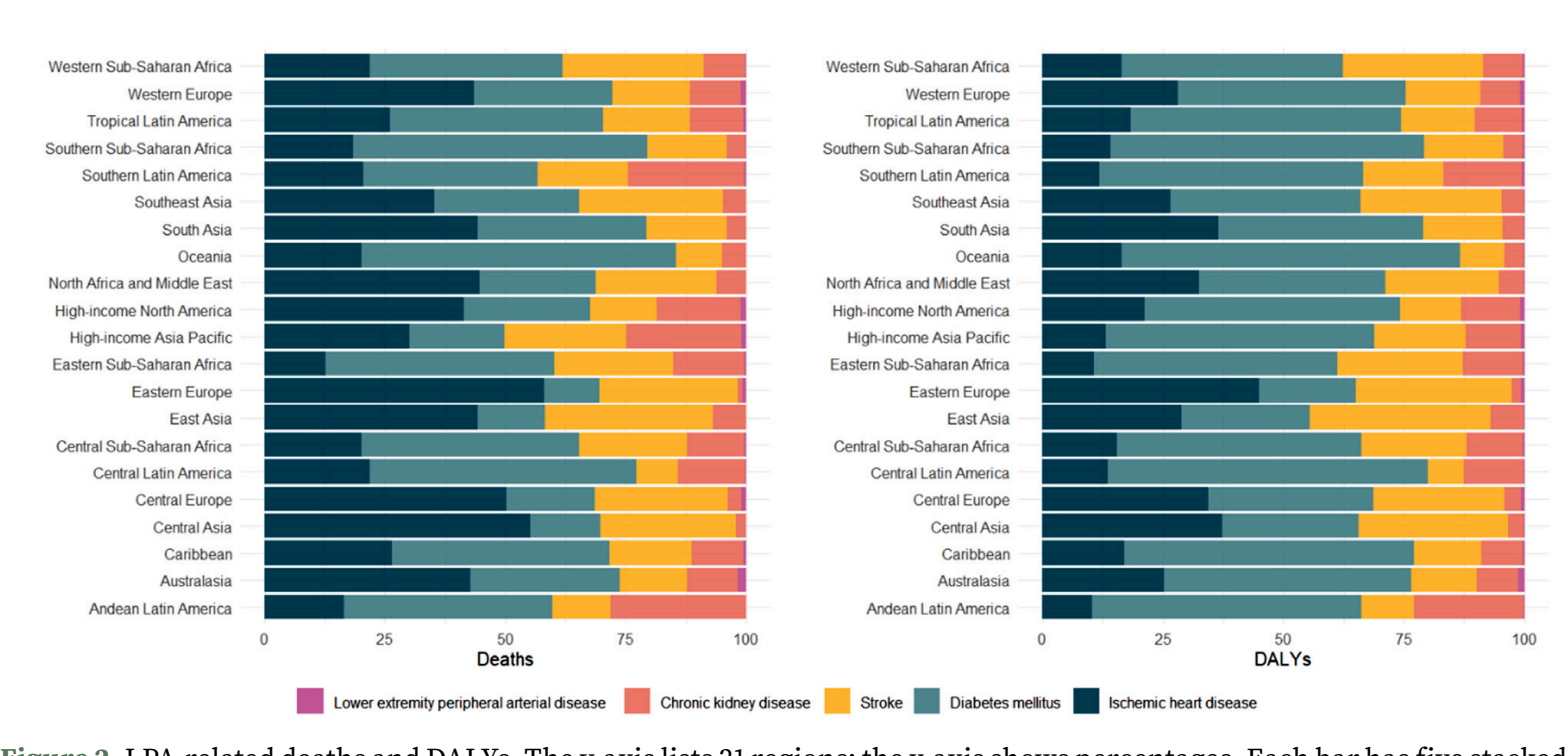
LPA-related deaths and DALYs. The y-axis lists 21 regions; the x-axis shows percentages. Each bar has five stacked segments, colour-coded for LEPAD (light pink), CKD (coral), stroke (gold), diabetes mellitus (grey-blue), and IHD (dark teal), indicating disease burden proportions. CKD – chronic kidney disease, DALYs – disability-adjusted life years, IHD – ischemic heart disease, LEPAD – lower extremity peripheral artery disease, LPA – low physical activity.

Based on the 2021 disease burden across 21 regions, the LPA-related death burden of IHD dominated in most of them, especially in Eastern Europe (~ 20 000 deaths), Central Europe (~ 8800 deaths), and Central Asia (~ 2400 deaths) (**Figure 2**, Table S3 in the **Online Supplementary Document**). Moreover, the LPA-related death burden of diabetes mellitus was also a major threat, ranking as the second leading cause of death, with ~ 7715 deaths in Central America (**Figure 2**, **Table 2**). LPA-related stroke deaths were evenly distributed, *e.g.* 46 112 in East Asia (**Figure 2**, Table S3 in the **Online Supplementary Document**). The distribution of LPA-related CKD deaths resembled that of stroke, *e.g.* 553 deaths in Southern Latin America (**Figure 2**, **Table 3**). LPA-related LEPAD deaths were the lowest, with even Western Europe, the region most affected by LPA, having only 464 deaths (**Figure 2**, Table S2 in the **Online Supplementary Document**). In terms of 2021 LPA-related DALYs, diabetes dominated in most regions, especially in Central and Southern Latin America (~ 240 000 disability cases) and Oceania (~ 18 000 disability cases) (**Figure 2**, **Table 2**), and Eastern Europe had a high proportion of IHD (~ 270 000 disability cases). Stroke and CKD also had similar distribution patterns, and LEPAD had the lowest DALYs value (**Figure 2**).

**Table 2:** Global death and DALYs associated with LPA caused by diabetes mellitus (1990–2021)*

	Death					DALYs				
	**1990**		**2021**		**1990 − 2021**	**1990**		**2021**		**1990 − 2021**
	**n (95% UI)**	**ASMRs (95% UI)**	**n (95% UI)**	**ASMRs (95% UI)**	**EAPCs (95% UI)**	**n (95% UI)**	**ASDRs (95% UI)**	**n (95% UI)**	**ASDRs (95% UI)**	**EAPCs (95% CI)**
**Location**										
Global	55 146 (23 698, 84 352)	5.94 (2.56, 9.09)	147 907 (63 453, 228 403)	6.52 (2.8, 10.07)	0.25 (0.2, 0.31)	1 664 981 (712 714, 2 595 137)	161.99 (69.42, 252.53)	5 238 421 (2 280 033, 8 294 823)	222.4 (96.82, 351.97)	0.92 (0.87, 0.96)
High-SDI	14 672(6207, 23 123)	4.78 (2.02, 7.54)	21 292 (8796, 33 630)	3.18 (1.32, 5.05)	−1.65 (−1.86, −1.45)	414 898 (174 454, 666 907)	135.82 (57.06, 218.49)	1 011 220 (426 110, 1 678 854)	175.72 (73.82, 293.77)	0.6 (0.48, 0.72)
High-middle SDI	10 768 (4606, 16 803)	4.66 (2.01, 7.26)	25 116 (10 812, 39 773)	4.72 (2.03, 7.47)	0.05 (−0.03, 0.13)	335 371 (141 580, 532 778)	129.74 (54.98, 205.36)	913 448 (387 401, 1 482 618)	166.58 (70.62, 270.33)	0.72 (0.66, 0.77)
Middle-SDI	15 932 (6828, 24 633)	7.36 (3.17, 11.36)	55 122 (23 913, 85 080)	8.42 (3.67, 12.99)	0.42 (0.36, 0.48)	511 249 (218 859, 806 395)	199.43 (85.79, 313.28)	1 893 924 (819 421, 2 983 607)	261.88 (113.62, 411.54)	0.77 (0.72, 0.81)
Low − middle SDI	10 234 (4237, 16 113)	7.96 (3.31, 12.47)	37 475 (16 054, 59 275)	11.7 (4.99, 18.46)	1.39 (1.31, 1.46)	302 423 (126 594, 481 948)	201.77 (84.85, 320.28)	1 140 700 (4 889 62, 1 825 978)	312.21 (133.77, 498.38)	1.44 (1.4, 1.48)
Low-SDI	3448 (1415, 5568)	7.4 (3.03, 11.92)	8719 (3633, 14 002)	8.47 (3.49, 13.58)	0.48 (0.38, 0.59)	98 217 (40 336, 159 563)	178.01 (73.34, 288.17)	272 485 (114 292, 438 600)	220.27 (91.98, 353.53)	0.64 (0.58, 0.71)
Andean Latin America	238 (89, 426)	4.81 (1.81, 8.54)	920 (340, 1654)	5.98 (2.21, 10.73)	0.62 (0.48, 0.76)	6785 (2464, 12 309)	128.98 (47.34, 232.34)	30 073 (11 127, 54 445)	190.24 (70.49, 343.49)	1.18 (1.09, 1.27)
Australasia	322 (130, 529)	5.16 (2.09, 8.49)	664 (270, 1090)	3.94 (1.6, 6.49)	−1.19 (−1.51, −0.88)	9011 (3568, 15 157)	140.03 (55.3, 235.96)	24 339 (9866, 41 740)	163.96 (66.16, 283.22)	0.32 (0.23, 0.42)
Caribbean	1101 (453, 1740)	16.8 (6.96, 26.5)	1951 (821, 3215)	13.1 (5.51, 21.58)	−0.93 (−1.02, −0.85)	30 843 (12 606, 49 569)	445.49 (182.44, 714.96)	68 918 (28 689, 114 546)	464.64 (193.59, 771.98)	0.01 (−0.05, 0.07)
Central Asia	221 (89, 361)	1.86 (0.75, 3.04)	645 (260, 1116)	3.31 (1.35, 5.66)	1.71 (1.31, 2.11)	8568 (3425,14 267)	68.83 (27.68, 114.26)	28 679 (11 294, 50 341)	132.7 (52.97, 231.07)	2 (1.74, 2.25)
Central Europe	1519 (640, 2433)	3.87 (1.63, 6.19)	3244 (1364, 5099)	4.87 (2.05, 7.67)	0.98 (0.8, 1.16)	56 213 (23 890, 92 259)	137.51 (58.37, 225.21)	119 606 (50 600, 195 594)	189.2 (79.52, 310.61)	1.17 (1.09, 1.26)
Central Latin America	2464 (918, 4274)	12.59 (4.71, 21.67)	7715 (3057, 13335)	11.85 (4.72, 20.43)	−0.4 (−0.71, −0.09)	72 560 (26 884 128 533)	337.71 (125.88, 593.56)	249 925 (98 706, 441 589)	366.47 (145.16, 645.44)	0.02 (−0.28, 0.31)
Central Sub−Saharan Africa	565 (209, 1062)	13.77 (5.14, 25.21)	1396 (493, 2691)	14.1(5.16, 26.57)	−0.03 (−0.13, 0.06)	15 140 (5564, 28 587)	297.38 (110.99, 548.85)	41 574 (14 612, 79 446)	332.76 (121.71, 622.95)	0.28 (0.18, 0.37)
East Asia	6108 (2331, 10 512)	3.45 (1.36, 5.84)	18 496 (7553, 32 436)	3.42 (1.41, 5.94)	−0.12 (−0.39, 0.16)	237 791 (87 492, 430 325)	110.06 (41.72, 195.81)	763 865 (287 814, 1 394 730)	126.98 (48.34, 230.05)	0.22 (0.07, 0.37)
Eastern Europe	697 (275, 1194)	0.92 (0.37, 1.58)	3984 (1566, 6809)	3.94 (1.55, 6.76)	3.87 (2.3, 5.47)	36 837 (13 805, 65 650)	48.23 (18.11, 85.61)	123 766 (46 967, 216 724)	123.75 (46.83, 217.68)	2.67 (2.14, 3.2)
Eastern Sub−Saharan Africa	985 (373, 1693)	6.29 (2.41, 10.7)	2043 (778, 3486)	6.04 (2.28, 10.32)	−0.32 (−0.39, −0.25)	25 870 (9673, 44 969)	140.87 (53.05, 242.16)	56 378 (21 797, 96 383)	139.54 (53.59, 237.95)	−0.22 (−0.29, −0.14)
High − income Asia Pacific	1740 (681, 2956)	3.37 (1.31, 5.73)	2330 (914, 4021)	1.44 (0.56, 2.52)	−2.83 (−3.04, −2.61)	80 623 (31 368, 138 135)	145.44 (56.59, 249.27)	209 124 (80 777, 373 581)	178.38 (67.67, 321.44)	0.49 (0.37, 0.62)
High-income North America	4506 (1668, 7973)	4.43 (1.63, 7.87)	6980 (2628, 12 086)	3.58 (1.34, 6.23)	−1.32 (−1.74, −0.9)	123 972 (41 802, 232 276)	126.02 (41.86, 238.84)	369 225 (137 609, 675 868)	201.22 (74.47, 372.23)	1.28 (1.14, 1.42)
North Africa and Middle East	3781 (1594, 5989)	10.55 (4.48, 16.72)	13 026 (5596, 20 663)	12.93 (5.55, 20.41)	1.11 (0.92, 1.29)	123 431 (52 544, 195 189)	291.08 (124.47, 459.15)	575 864 (245 497, 926 062)	480.18 (205.6, 769.39)	1.86 (1.75, 1.96)
Oceania	190 (74, 339)	28.2 (11.04, 49.5)	535 (210, 948)	30.86 (12.18, 54.1)	0.23 (0.14, 0.33)	5962 (2356, 10 573)	737.45 (290.55, 1295.76)	18 843 (7432, 33 518)	896.62 (355.62, 1582.93)	0.58 (0.49, 0.68)
South Asia	9304 (3727, 15 585)	8.16(3.31, 13.49)	38 226 (16 379, 62 419)	11.93 (5.09, 19.31)	1.3 (1.14, 1.46)	271 864 (108 521, 461 882)	198.97 (80.56, 333.95)	1 069 251 (451 039, 1 789 093)	289.6 (122.53, 480.58)	1.15 (1.04, 1.26)
Southeast Asia	4773 (1939, 7709)	8.3 (3.41, 13.42)	16 628 (7165, 26 386)	10.86 (4.72, 17.16)	0.8 (0.72, 0.87)	146 000 (59 600, 236 537)	226.96 (93.42, 366.78)	573 539 (239 931, 931 026)	335.38 (141.89, 540.78)	1.18 (1.15, 1.22)
Southern Latin America	545 (204, 1016)	4.63 (1.74, 8.58)	827 (291, 1478)	3.3 (1.16, 5.92)	−1.14 (−1.33, −0.94)	14 462 (5285, 27 652)	116.21 (42.66, 221.55)	31 708 (11 039, 58 872)	130.88 (45.45, 243.87)	0.24 (0.14, 0.34)
Southern Sub−Saharan Africa	1232 (512, 2004)	19.6 (8.21, 31.76)	4637 (1890, 7500)	35.93 (14.72, 57.92)	2.23 (1.78, 2.67)	32 183 (13 273, 52 870)	466.87 (194.16, 763.12)	124 533 (50 344, 203 634)	849.34 (345.71, 1382.51)	2.15 (1.8, 2.5)
Tropical Latin America	2902 (1151, 4890)	14.12 (5.69, 23.46)	8259 (3232, 13 841)	12.32 (4.84, 20.56)	−0.32 (−0.48, −0.15)	86 983 (33 505, 150 771)	374.65 (146.68, 640.52)	265 143 (100 324, 464 852)	380.82 (145.05, 664.51)	0.09 (0.02, 0.16)
Western Europe	10 471 (4518, 16 297)	6.22 (2.68, 9.7)	11 480 (4866, 18 170)	3.42 (1.45, 5.41)	−2 (−2.13, −1.86)	239 913 (103 299, 376 093)	145.29 (62.38, 228.29)	374 367 (162 266, 611 436)	140.23 (60.2,230.49)	−0.28 (−0.36, −0.19)
Western Sub−Saharan Africa	1484 (581, 2560)	8 (3.12, 13.71)	3921 (1521, 6563)	10.03 (3.9, 16.68)	0.75 (0.69, 0.81)	39 973 (15 636, 68 568)	184.86 (72.48, 315.06)	119 701 (47 187, 199 047)	250.91 (98.97,415.13)	0.99 (0.95, 1.03)

ASDRs – age-standardised death rates, ASMRs– age-standardised mortality rates, CI – confidence interval, DALYs – disability-adjusted life years, EAPCs – estimated annual percentage changes, LEPAD – lower extremity peripheral artery disease, UI – uncertainty interval

**Table 3:** Global death and DALYs of low physical activity caused by chronic kidney disease (1990–2021)

	Death					DALYs				
	**1990**		**2021**		**1990 − 2021**	**1990**		**2021**		**1990 − 2021**
	**n (95% UI)**	**ASMRs (95% UI)**	**n (95% UI)**	**ASMRs (95% UI)**	**EAPCs (95% CI)**	**n (95% UI)**	**ASDRs (95% UI)**	**n (95% UI)**	**ASDRs (95% UI)**	**EAPCs (95% CI)**
**Location**										
Global	13 435 (4879, 24 740)	1.51 (0.55, 2.78)	40145 (14974, 73094)	1.8 (0.67, 3.28)	0.64 (0.56, 0.72)	330 374 (119 650, 611 690)	33.4 (12.15, 61.64)	856 532 (313 039, 1 565 898)	36.99 (13.55, 67.56)	0.37 (0.31, 0.44)
High-SDI	3439 (1218, 6433)	1.15 (0.41, 2.16)	11530 (4429, 21244)	1.65 (0.63, 3.04)	1.35 (1.23, 1.47)	77 527 (27 646, 143 825)	25.7(9.14, 47.8)	203 369 (77 027, 370 083)	32.83 (12.35, 59.67)	0.9(0.82, 0.99)
High-middle SDI	2787 (998, 5161)	1.3 (0.47, 2.39)	7560 (2749, 13651)	1.45 (0.53, 2.62)	0.38 (0.27, 0.5)	68 466 (24 370, 127 577)	28.09 (10.1, 52.1)	155 404 (56 989, 282 541)	28.92 (10.62, 52.56)	0.13 (0.02, 0.25)
Middle-SDI	4188 (1501, 7688)	2.05 (0.74, 3.71)	13522 (4921, 24611)	2.12 (0.78, 3.84)	0.15 (0.09, 0.21)	105 848 (37 478, 198 075)	42.96 (15.37, 79.46)	309 425 (111 252, 569 125)	43.93 (15.88, 80.41)	0.12 (0.03, 0.2)
Low-middle SDI	2110 (733, 4008)	1.67 (0.59, 3.14)	5704 (2033, 10862)	1.74 (0.63, 3.29)	0.15 (0.09, 0.22)	55 238 (19 354, 105 728)	37.6 (13.37, 71.23)	142 985 (49 624, 275 321)	38.96 (13.7, 74.4)	0.11 (0.04, 0.17)
Low-SDI	896 (300, 1735)	1.96 (0.67, 3.76)	1793(592, 3473)	1.77 (0.6, 3.41)	−0.33 (−0.46, −0.21)	22 915(7685, 44 261)	42.42 (14.46, 80.92)	44 558 (14 625, 86 908)	36.77 (12.29, 70.94)	−0.54(−0.62, −0.46)
Andean Latin America	176 (59, 341)	3.66 (1.24, 7.03)	595(206, 1133)	3.85 (1.33, 7.31)	0.05 (−0.25, 0.36)	3599 (1175, 7096)	69.7 (23.04, 136.08)	12 246 (4152, 23 591)	77.07 (26.23, 148.04)	0.25(−0.04, 0.53)
Australasia	60 (19, 118)	1.01 (0.33, 1.99)	223(76, 437)	1.28 (0.43, 2.5)	1.34 (1.08, 1.6)	1371 (473, 2688)	21.94 (7.58, 43.09)	4104 (1481, 7896)	25.68 (9.24, 49.48)	0.86 (0.66, 1.07)
Caribbean	160 (61, 285)	2.54 (0.97, 4.53)	460(172, 814)	3.07 (1.14, 5.42)	1.23 (1.02, 1.44)	3674 (1381, 6615)	54.38 (20.51, 97.83)	9770 (3596, 17 428)	65.53 (24.12, 116.8)	1.17 (0.99, 1.35)
Central Asia	24 (7, 53)	0.21 (0.06, 0.45)	89 (27, 181)	0.47 (0.15, 0.95)	2.28 (1.85, 2.7)	1552 (501, 3133)	12.92 (4.17, 26.17)	3310 (1093, 6517)	15.75 (5.29, 30.89)	0.45 (0.23, 0.67)
Central Europe	343 (118, 650)	0.94 (0.32, 1.77)	510 (183, 947)	0.78 (0.28, 1.45)	−0.61 (−0.83, −0.39)	9167 (3214, 17009)	23.53 (8.27, 43.67)	11 993 (4347, 22 111)	19.22 (6.9, 35.58)	−0.6(−0.72, −0.48)
Central Latin America	427 (140, 798)	2.33 (0.78, 4.34)	1961 (648, 3857)	2.96 (0.98, 5.79)	1.29 (0.84, 1.74)	10 236 (3365, 19 347)	49.43 (16.42, 92.94)	46 867 (15 136, 93 190)	68.09 (22.12, 134.75)	1.48 (1.03, 1.92)
Central sub-Saharan Africa	185 (55, 387)	4.63 (1.45, 9.39)	367(106, 794)	3.76 (1.11, 8.04)	−0.89 (−1.01, −0.77)	4813 (1445, 10 151)	94.52 (29.38, 194.35)	9547(2711, 20 535)	75.62 (22.23, 159.95)	−0.95(−1.06, −0.84)
East Asia	3185 (1102, 6145)	2.06 (0.74, 3.89)	9176 (3182, 17 156)	1.78 (0.63, 3.31)	−0.61 (−0.74, −0.47)	78 817 (26 194, 154 655)	40.18 (13.76, 77.33)	195 884 (67 058, 371 876)	34.21 (11.83, 64.72)	−0.54(−0.69, −0.39)
Eastern Europe	102 (29, 226)	0.14 (0.04, 0.31)	355(106, 710)	0.36 (0.11, 0.71)	3.08 (2.64, 3.51)	6221 (2019, 12 770)	8.74 (2.84, 17.84)	11 105(3584, 22 230)	11.25 (3.63, 22.59)	0.77 (0.64, 0.9)
Eastern sub-Saharan Africa	296 (90, 615)	1.93 (0.59, 3.96)	631 (199, 1293)	1.94 (0.61, 3.95)	−0.18 (−0.25, −0.1)	6846 (2078, 14 311)	37.93 (11.63, 78.43)	13 735 (4309, 28 429)	35.48 (11.2, 72.71)	−0.44(−0.52, −0.36)
High-income Asia Pacific	867 (307, 1654)	1.88 (0.66, 3.58)	2855 (1027, 5608)	1.48(0.53, 2.89)	−0.71 (−0.83, −0.6)	18 195 (6490, 34 478)	35.51 (12.65, 67.41)	43 506 (15 449, 83 454)	27.72 (9.81, 53.01)	−0.67(−0.8, −0.54)
High-income North America	1065 (350, 2100)	1.06 (0.35, 2.09)	4653 (1735, 8489)	2.36 (0.88, 4.3)	2.68 (2.48, 2.87)	23 939 (7989, 46 850)	24.47 (8.1, 48.12)	86 190 (31 818, 158 317)	46.61 (17.12, 85.88)	2.14 (1.96, 2.33)
North Africa and Middle East	1198 (399, 2360)	3.45 (1.17, 6.78)	3348 (1144, 6337)	3.34 (1.16, 6.29)	−0.1 (−0.24, 0.03)	28 297 (9418, 55 767)	68.96 (23.29, 135.45)	77 785 (26 091, 147 323)	66.6 (22.79, 125.36)	−0.12(−0.18, −0.06)
Oceania	14 (5, 26)	2.3 (0.88, 4.27)	40 (15, 73)	2.6(1, 4.67)	0.31 (0.22, 0.4)	409 (151, 775)	54.26 (20.54, 101.06)	1089 (399, 1999)	57.44 (21.74, 103.63)	0.11 (0.04, 0.18)
South Asia	1464 (483, 2886)	1.23 (0.41, 2.39)	4287 (1480, 8439)	1.29 (0.45, 2.5)	0.15 (0.03, 0.27)	41 411 (13 558, 83 011)	30.05(10, 59.33)	113 029 (38 393, 224 281)	30.27 (10.4, 59.52)	0.01(−0.07, 0.09)
Southeast Asia	882 (305, 1704)	1.62 (0.56, 3.1)	2649 (930, 5105)	1.76 (0.62, 3.35)	0.17 (0.13, 0.22)	23 136 (7953, 45 394)	36.73 (12.73, 71.15)	66 392 (23 370, 129 649)	38.62 (13.67, 74.56)	0.14 (0.12, 0.16)
Southern Latin America	306 (96, 616)	2.69 (0.85, 5.37)	553 (173, 1117)	2.2 (0.69, 4.44)	−0.28 (−0.71, 0.14)	6042 (1848, 12 338)	49.87 (15.38, 101.39)	9475 (2903, 19 254)	38.73 (11.83, 78.79)	−0.51(−0.87, −0.15)
Southern sub-Saharan Africa	100 (32, 196)	1.68 (0.55, 3.26)	291 (97, 554)	2.36 (0.8, 4.46)	1.3 (1.01, 1.58)	2869 (940, 5569)	42.05 (14.01, 81.07)	7860 (2630, 14 954)	53.51 (18.21, 100.97)	0.89 (0.68, 1.11)
Tropical Latin America	554 (194, 1030)	2.8 (0.99, 5.16)	2070 (743, 3818)	3.06 (1.1, 5.64)	0.3 (0.1, 0.49)	14 056 (4886, 26 399)	61.89 (21.82, 115.01)	46 176 (16 500, 86 597)	66.31 (23.73, 124.07)	0.14(−0.07, 0.34)
Western Europe	1635 (557, 3100)	1.01 (0.34, 1.91)	4196 (1530, 7985)	1.16 (0.42, 2.21)	0.9 (0.72, 1.08)	35 836 (12 587, 67 178)	21.94 (7.66, 41.36)	64 816 (23 718, 122 274)	20.56 (7.46, 38.91)	−0.01(−0.11, 0.09)
Western sub-Saharan Africa	392 (130, 790)	2.24 (0.76, 4.42)	836 (272, 1655)	2.21 (0.74, 4.31)	−0.21 (−0.31, −0.11)	9885 (3247, 19 771)	47.59 (15.89, 93.8)	21 654 (7100, 42 771)	46.21 (15.6, 89.87)	−0.24(−0.33, −0.16)

ASDRs – age-standardised death rates, ASMRs– age-standardised mortality rates, CI – confidence interval, DALYs – disability-adjusted life years, EAPCs – estimated annual percentage changes, LEPAD – lower extremity peripheral artery disease, UI – uncertainty interval

**Figure 3:**
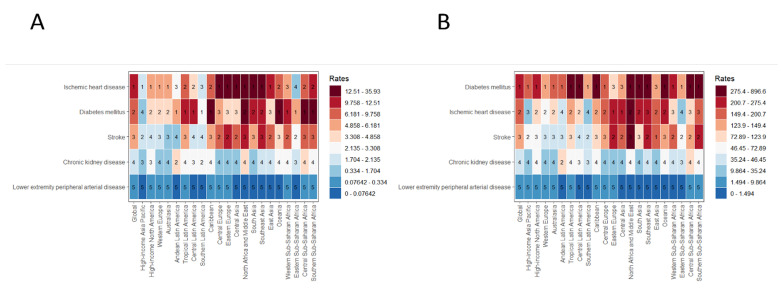
Heatmaps illustrate the disease burdens related to LPA of IHD, diabetes mellitus, stroke, CKD, and LEPAD across 22 global regions. Numbers 1–5 in each cell rank disease severity (1 = highest). The left heatmap shows ASR, and the right one shows ASMRs. ASR – age-standardised rates, ASMRs – age-standardised mortality rates, CKD – chronic kidney disease, IHD – ischemic heart disease, LEPAD – lower extremity peripheral artery disease, LPA – low physical activity.

LPA-related ASDRs for each disease showed significant geographical differences and a U-shaped relationship with SDI (**Figure 3**, Panel A). Central Asia had the heaviest IHD burden, while Central Asia, North Africa, and the Middle East had a high diabetes burden. Eastern Europe had a notably higher stroke burden. In contrast, the high-income Asia-Pacific region had a lower IHD burden, East Asia a lighter diabetes mellitus burden, and Australia a smaller stroke burden. These patterns show that LPA-related ASDRs for each disease decreased from the highest as SDI went from 0 to 0.5, then increased as SDI rose from 0.5 to 1 (**Figure 3**, Panel A). In terms of LPA-related ASMRs, North Africa and the Middle East had the highest IHD burden, Oceania led in diabetes mellitus burden, and North Africa and the Middle East had a notably higher stroke incidence. Meanwhile, the high-income Asia-Pacific region did well in IHD and stroke, while Eastern Europe had a lower diabetes mellitus burden. These data show that LPA-related ASMRs also have a U-shaped relationship with SDI, with obvious regional differences in disease burden (**Figure 3**, Panel B).

Furthermore, we also analysed the clear U-shaped graphs of regions for five diseases (IHD, stroke, LEPAD, diabetes mellitus, CKD) based on SDI level (Figure S1 in the **Online Supplementary Document**).

### Analysis of the burden of each LPA-related disease at the national level

The disease burden maps of 204 countries show significant geographical differences in LPA-related ASMRs relative to SDI. In high-SDI regions, such as Western Europe and North America, LPA-related ASMRs are generally lower than expected. Switzerland (65 per 100 000 people), Japan (72 per 100 000), and Norway (68 per 100 000) have the lowest levels globally. In contrast, low-SDI regions such as sub-Saharan Africa and South Asia have notably higher ASMRs, with the Central African Republic (312 per 100 000), Chad (298 per 100 000), and Niger (287 per 100 000) bearing the highest burden. Notably, some middle-SDI countries in Eastern Europe show an inverse relationship between LPA-related ASMRs and SDI ). The trends of LPA-related ASDRs reveal deeper contradictions. While high-SDI countries keep a low baseline, countries like Australia (+1.2% EAPCs), Canada (+0.9% EAPCs), and the USA (+0.7% EAPCs) saw upward trends from 1980 to 2020. In low-SDI regions like Rwanda (−2.1% EAPCs) and Ethiopia (−1.8% EAPCs), significant decreases occurred due to expanded basic medical coverage. Finally, LPA-related ASDRs in East Asian countries show a divergent trend (Figure S2 in the **Online Supplementary Document**).

### Age and sex patterns

Based on 2021 GBD data, a subgroup analysis of five LPA-related diseases on age and gender effects showed that in age-related analysis, LPA-related deaths significantly increased in those aged 60 years and older, especially for IHD and diabetes mellitus In the 60–89 age group, LPA-related DALYs rose sharply, with IHD, diabetes, and stroke being major causes of disability. Notably, in those 90 and older, LPA-related DALYs showed an inverse trend, likely due to reduced resistance and a higher prevalence of death as the main disease outcome. For 45–59-year-olds, LPA-related burden mainly manifested as DALYs. Gender analysis revealed that women had higher LPA-related deaths and DALYs across all age groups of the five diseases. In the 60–89 age group, there were ~ 150 000 LPA-related female deaths and 110 000 male deaths (Figure S3 in the **Online Supplementary Document**). These findings underscore the need for prevention strategies targeting the elderly, especially postmenopausal women vulnerable to LPA impacts.

### Analysis of the disease burden based on the SDI

From 1990 to 2021, the LPA-related death proportions of five diseases (IHD, diabetes mellitus, stroke, CKD, and LEPAD) changed globally and across five different SDI regions. Overall, the global and high-SDI LPA-related death proportions in IHD declined, while those in low and middle-SDI regions levelled off. Diabetes mellitus' LPA-related death proportions increased globally and at all SDI levels, especially in low and middle-SDI regions. Stroke's LPA-related death proportions decreased globally and across SDI levels. However, in 2021, IHD’s LPA-related death proportion remained dominant in all SDI regions. CKD's LPA-related death proportion slightly rose in all SDI regions, and LEPAD’s remained minimal (Figure S4 in the **Online Supplementary Document**). Over time, the LPA-related disease burden compositions in different SDI regions somewhat converged, though differences persisted.

From 1990 to 2021, LPA-related ASMRs and ASDRs for five diseases showed distinct trends across different SDI regions. In high and high-middle SDI regions (*e.g.* Europe, North America), medical advancements and more physical activity reduced LPA-related disease impacts. For instance, IHD’s LPA-related ASMRs dropped from 285 to 120 per 100 000. Conversely, low-SDI regions like sub-Saharan Africa, hampered by resource shortages, saw an 82% rise in diabetes’ LPA-related ASDRs. In middle-SDI areas such as Andean Latin America, diabetes’s LPA-related ASMRs had EAPCs of 0.62 (95% CI = 0.48, 0.76) from 1990–2021. High-SDI regions improved LPA-related burdens for IHD, stroke, and LEPAD, thanks to vascular interventions. However, middle- and low-SDI regions showed little progress, with IHD burdens levelling off (Figure S5 in the **Online Supplementary Document**). These trends expose global health disparities: high-SDI regions benefit from exercise culture, advanced medicine, and good policies, while middle- and low-SDI regions struggle in the ‛disease transition’. Addressing this call for targeted policies, better healthcare, more physical activity promotion, and international cooperation.

### Analysis of the APC model

The LPA-related DALYs ratios and mortality for these diseases showed distinct age and SDI differences, with both increasing with age, especially among individuals aged 60 years and older. Significantly, LPA-related disease burdens varied greatly across SDI regions. The LPA-related disease burdens of IHD, stroke, and LEPAD improved in high and high-middle-SDI regions, while low and middle-SDI regions should watch out for the growing harm of such burdens. Additionally, the rising LPA-related diabetes burden is a major issue everywhere, particularly in low and middle-SDI regions. CKD’s LPA-related burden showed a relatively steady, slow upward trend across SDI regions. Finally, in period and birth cohort analyses, the LPA-related risk ratios for IHD, stroke, and LEPAD shifted from >1 to <1 during 2007–11 and 1932–41, opposite to the trend of diabetes mellitus’ LPA-related risk ratios (Figure S6 in the **Online Supplementary Document**). This suggests these periods were likely key times for changes in attitudes towards exercise, medical progress, or socio-economic conditions.

Furthermore, we have also analysed the deaths (Figure S7 in the **Online Supplementary Document**) and DALYs (Figure S8 in the **Online Supplementary Document**) for five noncommunicable diseases (IHD, stroke, LEPAD, diabetes mellitus, CKD) in 5 different countries (USA, China, Egypt, India, Nigeria).

### The relationship between the SDI and the LPA-related disease burdens of these five diseases (decomposition analysis)

From 1990 to 2021, global LPA-related case growth data showed population factors accounted for 52% of the total increase, while epidemiological change contributed 37%. LPA-related cases grew by 68% in diabetes, 57% in CKD, and 49% in LEPAD. Diabetes mellitus in middle-SDI regions (*e.g.* Latin America) saw a +215% total change. In low-SDI regions (*e.g.* sub-Saharan Africa), population growth made up 58% of diabetes mellitus’ LPA-related case growth, and stroke’s LPA-related cases grew by +89%. Low- and middle-SDI regions’ LPA-related disease burdens strongly correlated with population growth, especially in less-regulated low- and middle-income countries, potentially leading to compounded health impacts. High-SDI regions differed: in Europe and North America, epidemiological change dominated LPA-related disease burdens for IHD, stroke, and LEPAD, showing exercise and medical advances mitigated LPA’s effects. Notably, ageing had a negligible impact on LPA-related disease burdens (Figure S9 in the **Online Supplementary Document**), emphasising the health risks of inactivity across all age groups.

## DISCUSSION

We found that from 1990 to 2021, the global burden of five LPA-related diseases (IHD, stroke, LEPAD, diabetes mellitus, and CKD) among people above 45 years old has increased considerably, especially among elderly women. The LPA-related disease burden varied across regions with different SDI levels. In particular, the burden of IHD remained relatively high in low and middle-SDI regions, while diabetes mellitus showed an increasingly significant impact on the global disease burden. The APC model indicated that since 2007–11, the risk ratios of LPA-related DALYs and deaths for IHD, stroke, and LEPAD have declined from >1 to <1. In contrast, diabetes mellitus shows the opposite trend, highlighting the continuous and substantial impact of LPA on diabetes-related morbidity. Decomposition analysis shows that demographic changes and epidemiological transitions are the main drivers of the LPA-related burden of the five diseases. In high-SDI regions, epidemiological change is dominant, while in low and middle-SDI regions, population growth is the key factor. The synergistic interaction of these factors with LPA is expected to considerably increase the future disease burden.

From 1990 to 2021, LPA-related deaths and DALYs in IHD, stroke, and LEPAD rose, while LPA-related ASMRs and ASDRs significantly declined. For instance, IHD’s LPA-related ASMRs dropped from 14.82 to 10.41 per 100 000 people, with an EAPC of −1.19%, and similar decreases occurred in stroke and LEPAD. These improvements likely stem from increased global participation in physical activity, enhanced vascular intervention techniques, better medical policies, and extensive prevention strategies implemented from 2007 to 2011, in line with prior research. However, no data on global activity trends were presented in this study, which needs to be clarified in future investigations. First, the Olympics and various international events have served as direct channels for the public to understand physical exercise [28]. Second, the number and availability of organised sports activities are growing worldwide, allowing people of all ages to participate and improve their fitness [29]. Such increases in public exercises could help alleviate the burden of LPA on stroke, IHD and LEPAD [17–19]. In 2011, Horst Neubauer and colleagues advocated for personalised aspirin-clopidogrel combination therapy for coronary heart disease patients for at least one year post-surgery, reducing adverse effects and restenosis risk while boosting long-term survival [30]. In 2007, Penumbra's FDA-approved aspiration device revolutionised stroke treatment, propelling interventional therapies forward [31]. Moreover, the same year witnessed rapid advancements in peripheral arterial disease intervention; stent placement notably decreased patient DALYs [32]. Meanwhile, effective vascular disease policies were introduced, such as the USA ‛Million Hearts’ initiative in 2011 which, by combining prevention and treatment strategies, halved new heart disease and stroke cases in five years [33]. At the population level, enhanced healthcare infrastructure has facilitated earlier diagnosis, effective management, and prevention of LPA-related burdens in IHD, stroke. and LEPAD [34], and public health initiatives promoting physical activity, healthy dietary patterns, and tobacco cessation have effectively reduced the associated risk factors [35–37]. Furthermore, global policy initiatives, including policies for actively participating in physical exercise, tobacco taxes, salt reduction efforts, and hypertension control programmes, have significantly alleviated these burdens [38–44]. At the individual level, tailored exercise plans for personal fitness, increased use of evidence-based medications, including antihypertensives, statins, and antithrombotic therapies, have substantially reduced LPA-related mortality and disability rates in IHD, stroke, and LEPAD [1,44–46]. Additionally, advancements in vascular disease treatment techniques, such as thrombolysis and mechanical thrombectomy for large vessel occlusion stroke, have notably enhanced patient outcomes [47]. The application of intra-aortic balloon pump and extracorporeal membrane oxygenation enables more high-risk IHD patients to safely undergo coronary interventions [48], while peripheral stenting reduces the mortality and DALYs of patients with LEPAD [49].

Furthermore, we noted a worrying upward trend in the age-standardised burden of LPA-related diabetes mellitus. From 1990 to 2021, global LPA-related ASMRs and ASDRs rose, with EAPCs of 0.25% and 0.92%, respectively. In the APC model, the LPA-related risk ratio in diabetes mellitus shifted from <1 to >1 for those born between 2007–11 and 1932–41. As a key risk factor for diseases like IHD, diabetes mellitus is closely linked to insufficient physical activity and metabolic disorders [19,50]. Despite the progress made in reducing the burdens related to LPA in IHD, stroke, and LEPAD through better healthcare and prevention measures, the growing burden of LPA-related diabetes mellitus threatens these achievements. If left unchecked, it could reverse overall progress. Therefore, urgent, targeted interventions for physical activity promotion and diabetes mellitus management are needed, and comprehensive strategies, including public health campaigns, lifestyle interventions, and improved healthcare access, are essential to curb LPA burdens of these diseases [51–53].

We found severe regional disparities in the LPA burden of the five diseases. Due to effective healthcare and prevention, high-SDI regions reduced LPA-related ASMRs and ASDRs for IHD, stroke, and LEPAD; however, low and middle-SDI regions saw stagnation or increases, particularly in IHD and diabetes mellitus. For instance, these regions had the largest LPA-related ASMRs increases for diabetes mellitus (low-SDI, EAPCs = 0.48%; low-middle SDI, EAPCs = 1.39%; middle-SDI, EAPCs = 0.42%) and stagnant IHD ASMRs (low-SDI, EAPCs = 0.08%, low-middle SDI, EAPCs = 0.22%, middle SDI, EAPCs = 0.05%). These differences likely result from variations in lack of physical activity, healthcare access, resource allocation, and public health priorities [17,19,54,55]. Regions with rapidly ageing populations and restricted healthcare infrastructure, such as Andean Latin America, Central Asia, North Africa and the Middle East, South Asia, and Southeast Asia, struggle to handle the growing LPA-burden of diabetes mellitus [56]. Moreover, lifestyle shifts, *e.g.* higher processed food intake, sedentary habits, and urbanisation have worsened the rising LPA-related burden of the five diseases in regions with middle- and low-SDI [57–59]. In contrast, high-SDI countries have reduced this burden thanks to better public health policies, improved healthcare, and healthy physical exercise habits [57–59]. These lessons can guide resource-constrained areas in tailoring interventions to their local contexts.

In age- and gender-based subgroup analysis, we found that the LPA-related burden of the five diseases significantly increased among individuals aged 60–89 years, particularly for IHD, diabetes mellitus, and stroke, resulting in 410 626 deaths and 9 288 607 DALYs, which aligns with previous studies [47,58]. Among women aged 60 years or older, LPA disease burden exceeded that of men, aligning with the loss of estrogen protection post-menopause [60–64]. This emphasises the need to boost elderly disease management and community care, especially for women. Using decomposition analysis, we found that global increases in the LPA-related disease burden among individuals over 45 years are driven by both population and epidemiological shifts. Regionally, the LPA-related disease burden in high-SDI regions is mainly shaped by epidemiological change, whereas population factors prevail in low- and middle-SDI regions. Thus, besides boosting public engagement in physical exercise, upgrading medical standards, and refining medical policies, middle- and low-SDI regions should efficiently enforce family planning policies to curb excessive population growth [65].

LPA is a significant risk factor for the onset and progression of CKD. Prolonged physical inactivity could lead to metabolic abnormalities such as obesity, insulin resistance, and hypertension, which increase the filtration burden on the kidneys and accelerate glomerulosclerosis. Moreover, LPA could reduce the body’s immunity, increase the risk of infection, and indirectly induce or exacerbate kidney damage. In high-SDI regions, well-established healthcare systems and exercise rehabilitation programmes could be provided to CKD patients. In low- and middle-SDI regions, priority should be given to strengthening CKD screening, while promoting low-cost and easily accessible forms of exercise, and simultaneously improving basic medical facilities. LPA could further aggravate vascular endothelial dysfunction, accelerate the process of atherosclerosis, and increase the risk of LEPAD. Conversely, LEPAD patients often actively reduce physical activity due to lower extremity pain. In high-SDI regions, specialised LEPAD rehabilitation centres could be established, equipped with vascular ultrasound and other equipment to assess the safety of exercise, and combined with drug therapy and exercise intervention. In low- and middle-SDI regions, emphasis should be placed on enhancing primary care physicians’ ability to identify LEPAD, while promoting low-cost exercise interventions suitable for local conditions and improving the allocation of medical resources.

Furthermore, our results showed that the burden of LPA-related diseases is on the rise, while the ASMRs and ASDRs of several diseases are declining. These two results may seem contradictory, but both are based on comprehensive analysis. Although age-standardised rates are decreasing, which may reflect the effectiveness of medical advances and interventions, the overall absolute burden is still increasing due to population growth and ageing.

### Limitations

First, the GBD study’s model-based estimations in regions lacking comprehensive vital registration and disease surveillance may introduce bias, especially in areas with insufficient data. Second, our focus on five LPA-related diseases does not fully capture their complex interactions with other risk factors; thus, individual-level data studies are needed for clarification. Third, our predictive analysis, which we conducted using projected population and epidemiological trends, may overlook the real-world influence of future factors. Fourth, although the GBD study attempted to isolate the impact of LPA through risk factor decomposition, we did not explore the effects caused by comorbid risk factors (*e.g.* diet, smoking, air pollution, *etc.*) [66–68]. Fifth, we excluded younger adults (*e.g.* 30–44 years old), which may underestimate the disease burden of LPA in high-risk populations. Meanwhile, we also did not assess the interaction between ageing trends (compression *vs.* expansion of morbidity) and low physical activity. Sixth, we oversimplified drivers of disease burden in our decomposition analysis by attributing changes solely to ‛population growth’ or ‛epidemiological shifts’. We did not consider structural determinants (*e.g.* urbanisation, healthcare access) and failed to explore how these factors modify LPA’s impact. Seventh, we recognise that a focussed analysis on urban-rural differences would have significantly enhanced the value of the research; however, the GBD database we relied on does not currently provide such data. Eighth, we did not prioritise interventions (*e.g.* workplace activity programmes and public awareness campaigns) based on evidence specific to the SDI. Finally, we failed to provide visual explanations for the complex relationships described, particularly the U-shaped association between the SDI and disease burden.

## CONCLUSIONS

We examined the global LPA-related disease burden of five conditions in adults aged 45 years and older, and analysed spatiotemporal trends. The overall burden is rising, mainly affecting the elderly, especially women. The growing LPA-related diabetes mellitus burden among those aged 45 years and older highlights the global struggle for glycemic control. IHD drives elderly death and DALYs, especially in low- and middle-SDI regions, demanding better vascular intervention. High-SDI areas have reduced the overall LPA-related burden via good exercise habits and better healthcare; however, it is still necessary to maintain regular physical exercise and pay attention to issues related to epidemiological change. In contrast, we found increased LPA-related burdens in low- and middle-SDI regions, needing more exercise promotion, healthcare improvement, policy adjustment, and family planning. Policymakers should boost public engagement in physical exercise initiatives and tailor interventions to local needs. Based on our findings, we provide decision-makers with key insights to mitigate LPA-related disease burden.

## Additional material


Online Supplementary Document
