# Supplementary material for: Low physical activity-related disease burden, 1990–2021: assessment of global trends and social determinants based on GBD 2021 data
Source: J Glob Health. 2025 Dec 5;15:04314. doi: 10.7189/jogh.15.04314 (PMC12677240; doi:10.7189/jogh.15.04314)
Supplement: Online Supplementary Document [file jogh-15-04314-s001.pdf]

Supplement to: Liu H, Liu Z, Gong Y, Guo J, Liu X, Sun Y, Tang W, Cheng W, Jin W. Low physical activity-related disease burden, 1990–2021: assessment of global trends and social determinants based on GBD 2021 data. J Glob Health. 2025;15:04314.

Figure S1

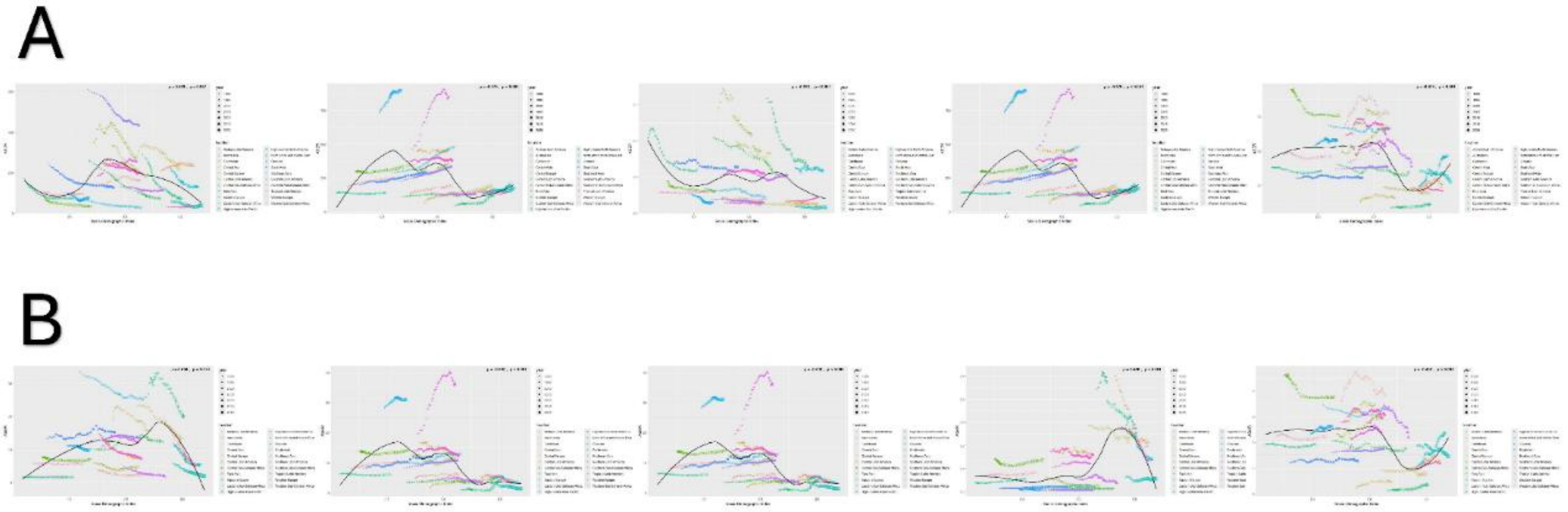

U-shaped graphs showing five diseases (IHD, stroke, LEPAD, diabetes mellitus, CKD) in five countries. A. ASDRs. B. ASMRs. IHD: ischemic heart disease, LEPAD: lower extremity peripheral artery disease, CKD: chronic kidney disease.

Figure S2

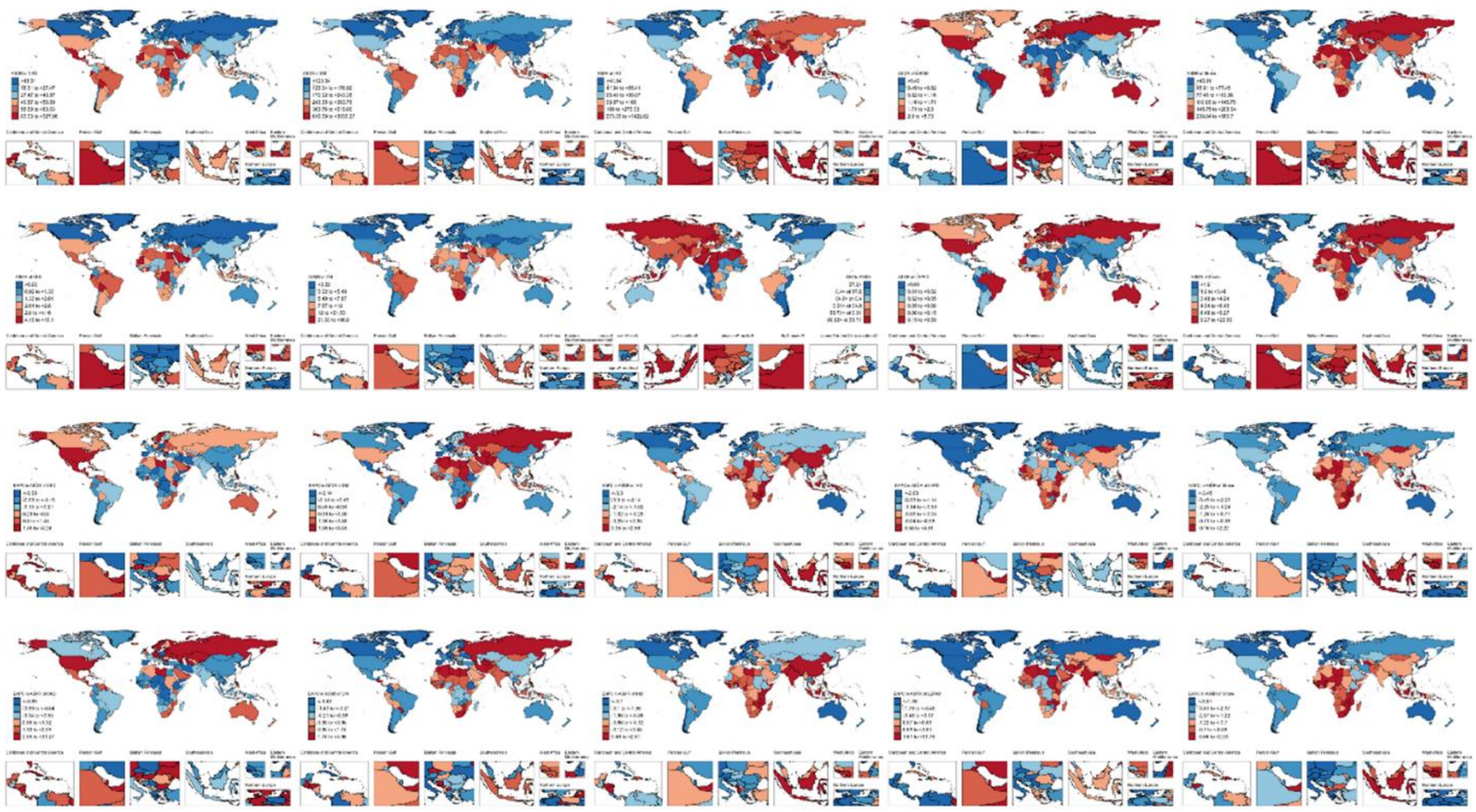

This world map illustrates the LPA-related disease burden of 204 countries. Each column represents a disease (eg, IHD, diabetes mellitus) and each line indicates different epidemiological indicators: ASDRs, ASMRs, and EAPCs. A blue-to-red color gradient shows value intensity, categorized into six levels in the right-side legend. Regional maps cover areas like the Caribbean, Persian Gulf, and Balkans. ASDRs: age-standardized disability rates, ASMRs: age-standardized mortality rates, EAPCs: estimated annual percentage change, LPA: low physical activity, IHD: ischemic heart disease.

Figure S3

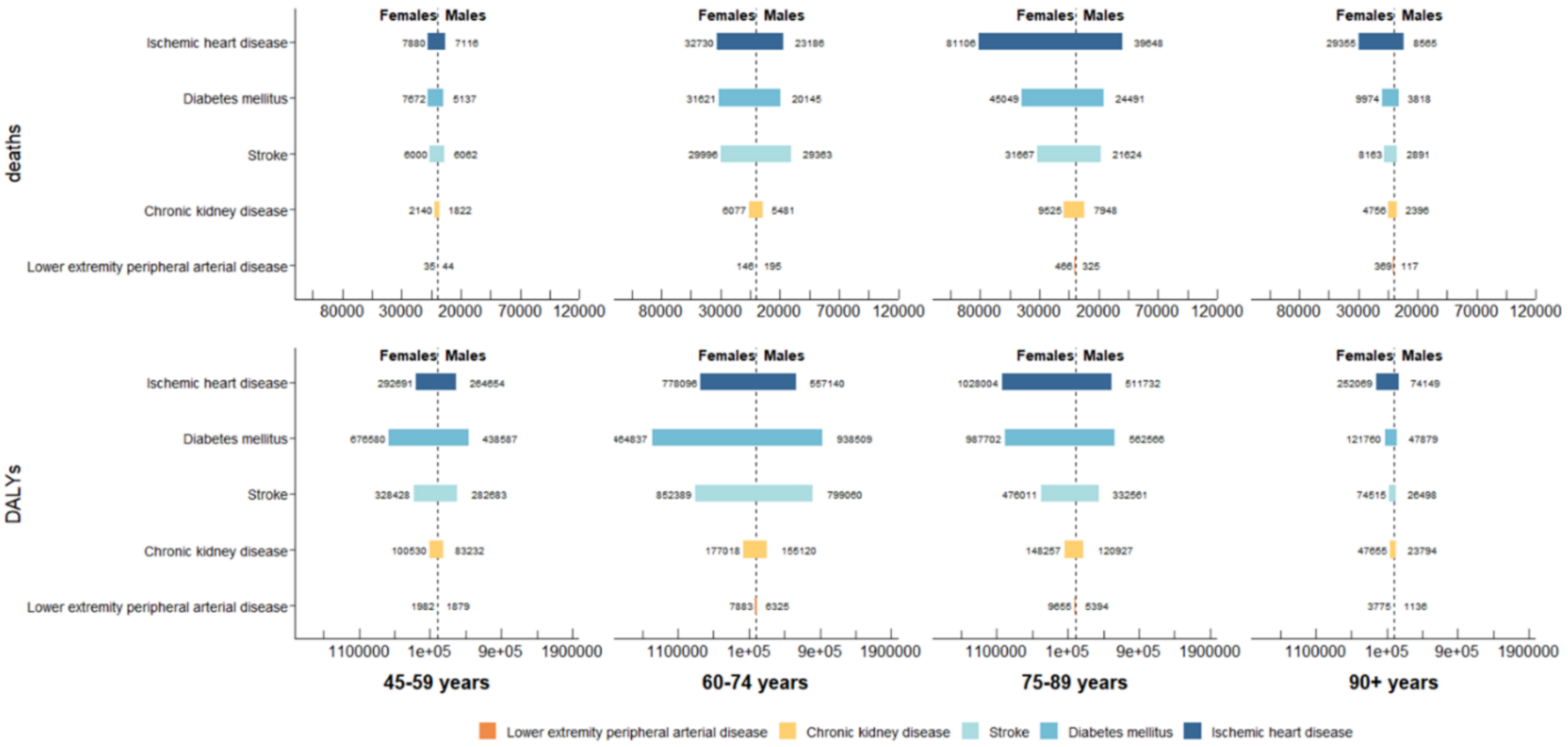

This figure shows LPA-related deaths and DALYs data across age groups (45-59 to 90+ years) and genders. For each age group, paired bar charts represent females (left) and males (right), with five diseases listed on the y-axis. Bars are color-coded by disease type (eg, diabetes mellitus, stroke).

Figure S4

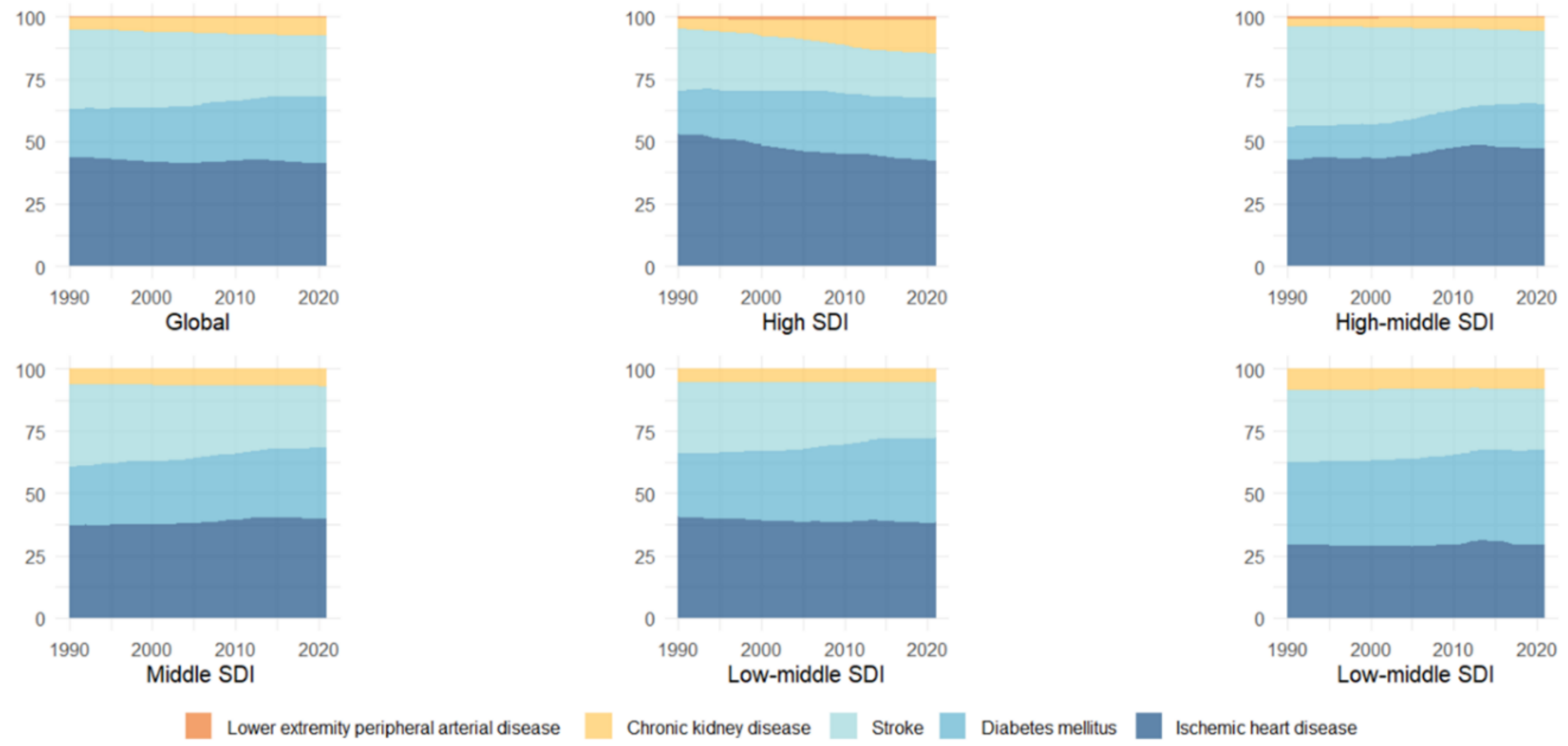

The figure comprises six stacked area charts, categorized by global and SDI regions. Color-coded for five diseases (eg, LEPAD, CKD), it shows the incidence proportion of patients from 1990 - 2021. The x-axis depicts how the area of each disease evolves over time. SDI: socio-demographic index, LEPAD: lower extremity peripheral artery disease, CKD: chronic kidney disease.

Figure S5

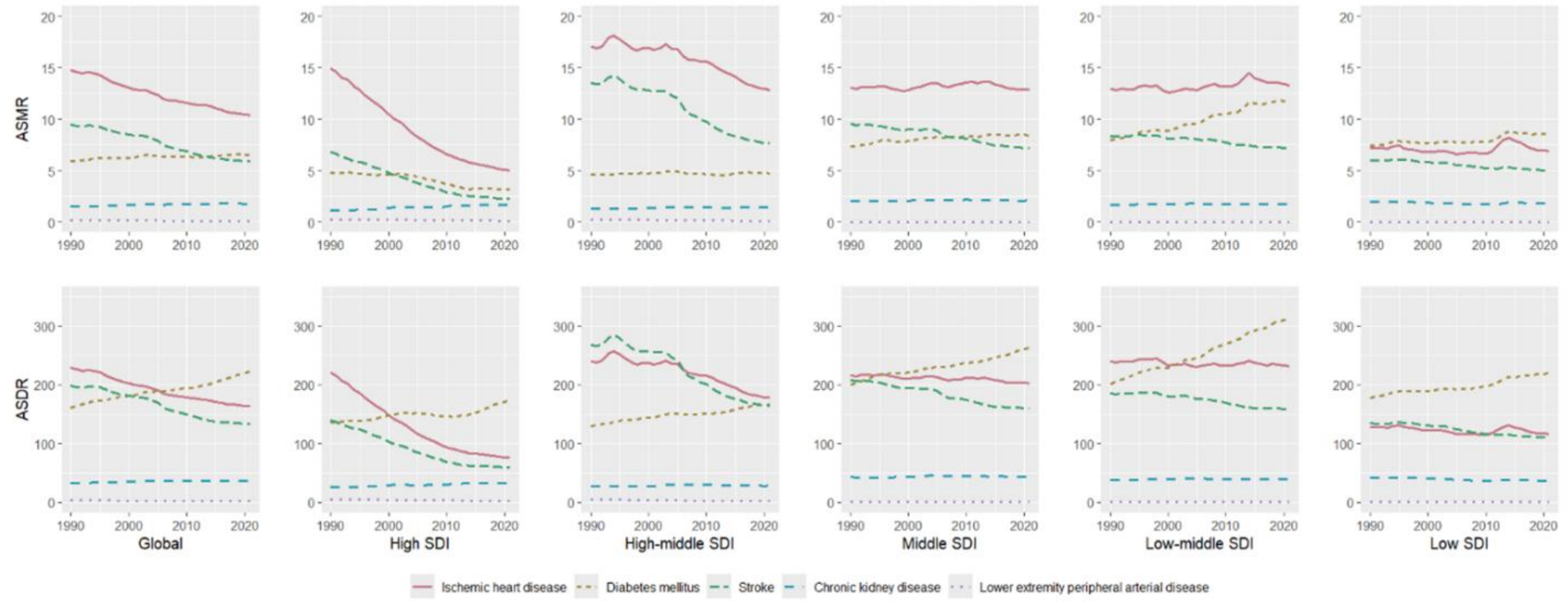

The figure’s first row shows LPA-related ASMRs, and the second row shows LPA-related ASDRs. Each column corresponds to the global and various SDI levels. Lines of different colors and types distinguish five diseases (eg, diabetes mellitus, stroke) in each graph. The y-axis represents ASMRs/ASDRs values, with the x-axis spanning four time points from 1990 to 2020. ASMRs: age-standardized mortality rates, ASDRs: age-standardized disability rates, SDI: socio-demographic index, LPA: low physical activity.

Figure S6

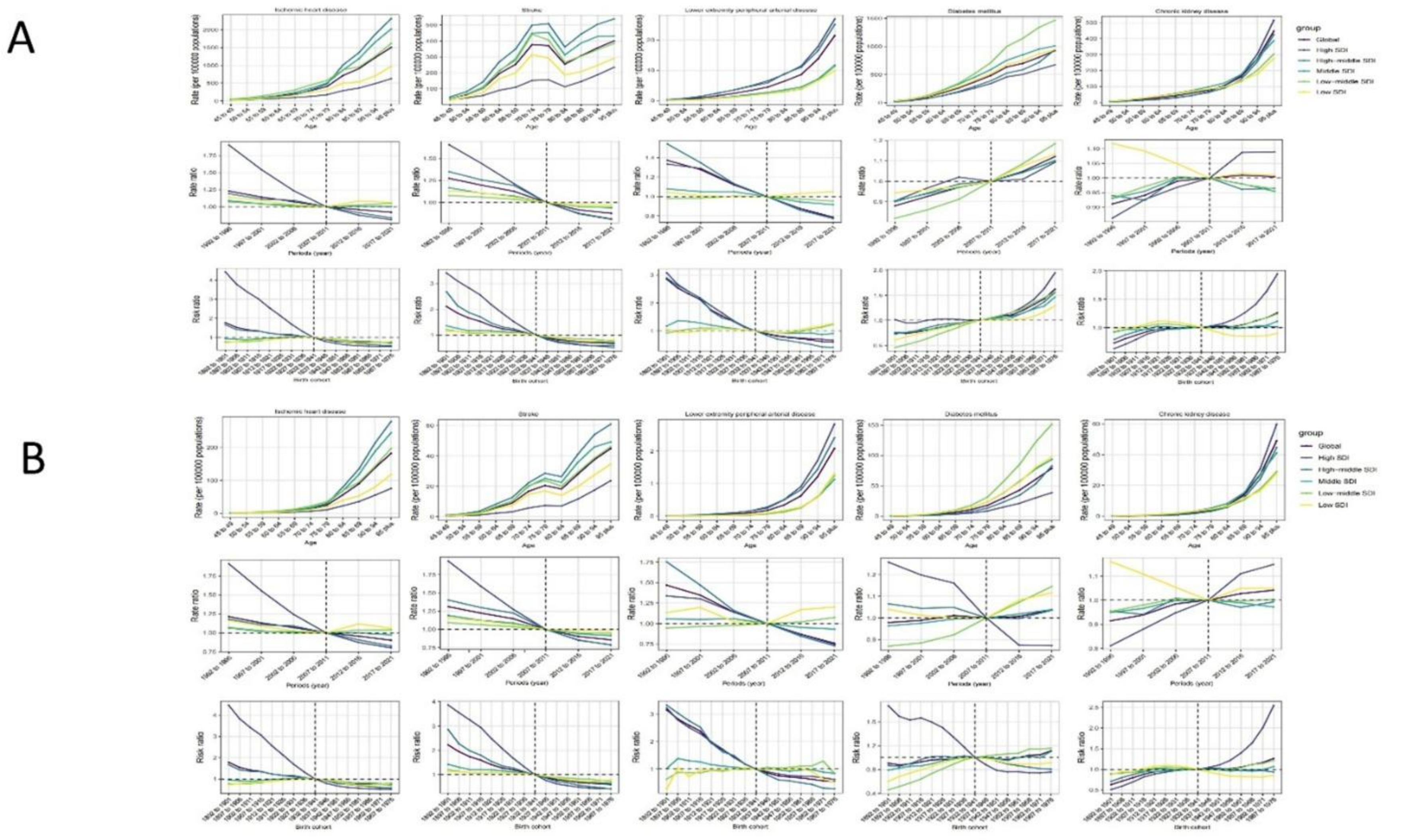

This figure presents the APC model results. Figure A illustrates LPA-related DALYs, and Figure B shows LPA-related deaths. Five diseases (such as IHD and diabetes mellitus) are lined up vertically in each panel. Each disease module contains three horizontal subgraphs: the age-stratified DALYs ratio/mortality rate line graph on the left, the period RR line graph in the middle, and the birth cohort RR line graph on the right. Different colors differentiate the global group from high to low-SDI groups. APC: age-period-cohort, LPA: low physical activity, DALYs: disability-adjusted life years, RR: risk ratio, SDI: socio-demographic index, IHD: ischemic heart disease.

Figure S7

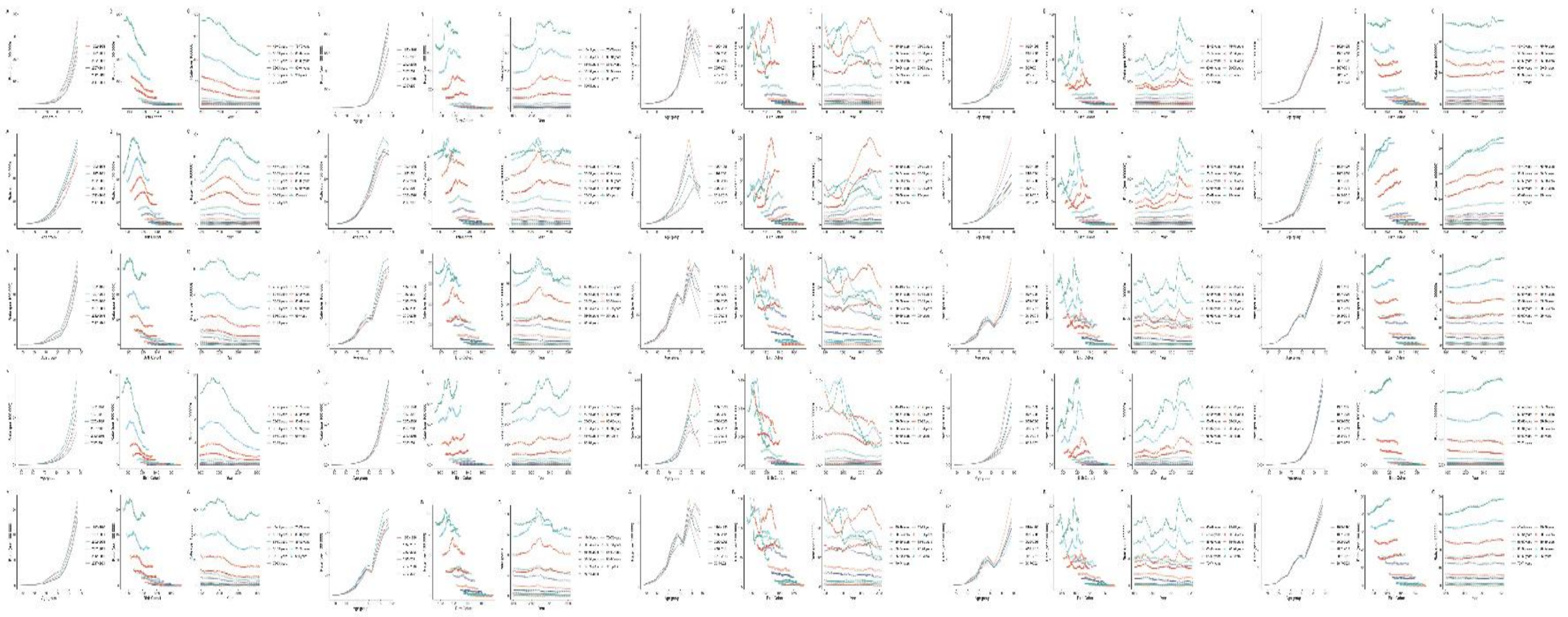

Death APC analysis of 5 diseases in different countries. From left to right are five representative countries: the United States, China, Egypt, India, and Nigeria (Africa). For each country, the APC graphs from top to bottom correspond to the following diseases: IHD, stroke, LEPAD, diabetes mellitus, and CKD. IHD: ischemic heart disease, LEPAD: lower extremity peripheral artery disease, CKD: chronic kidney disease.

Figure S8

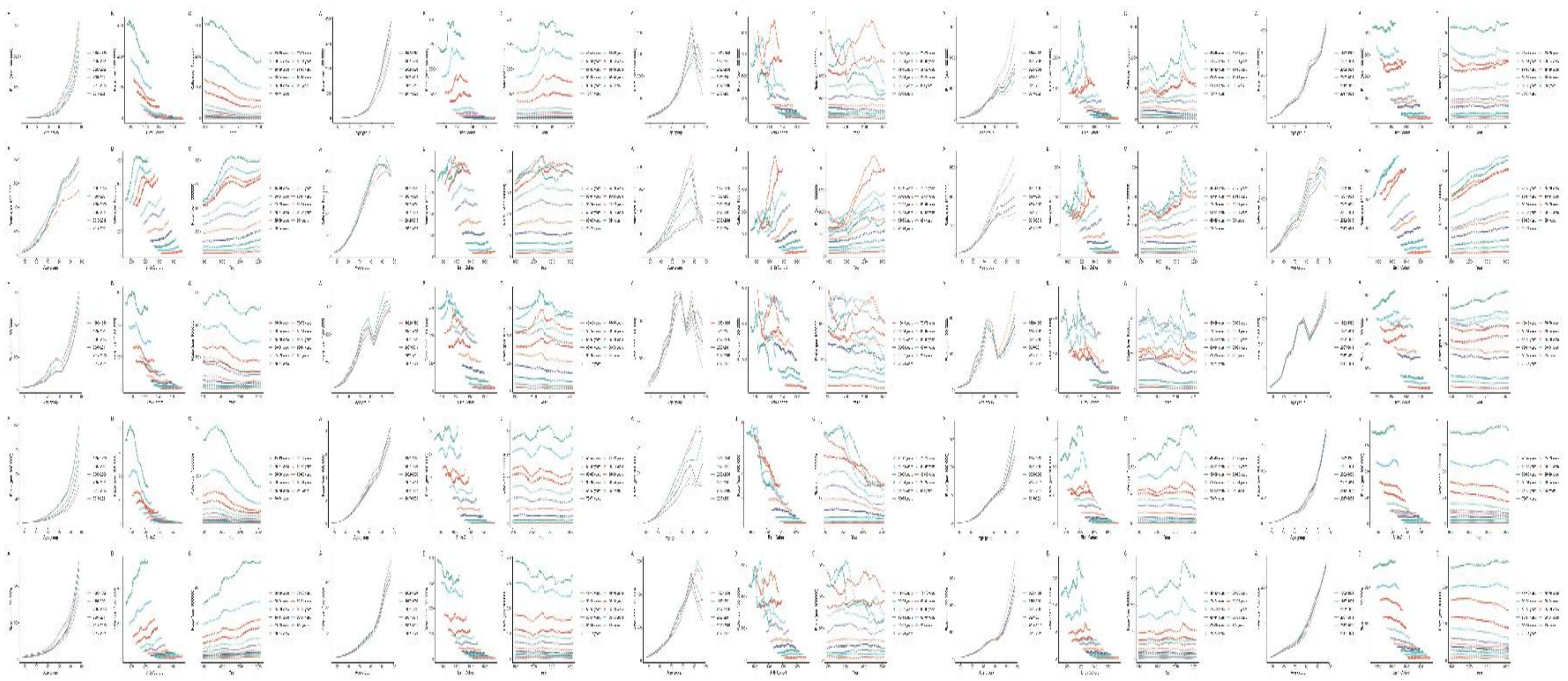

DALYs APC analysis of 5 diseases in different countries. From left to right are five representative countries: the United States, China, Egypt, India, and Nigeria (Africa). For each country, the APC graphs from top to bottom correspond to the following diseases: IHD, stroke, LEPAD, diabetes mellitus, and CKD. IHD: ischemic heart disease, LEPAD: lower extremity peripheral artery disease, CKD: chronic kidney disease.

Figure S9

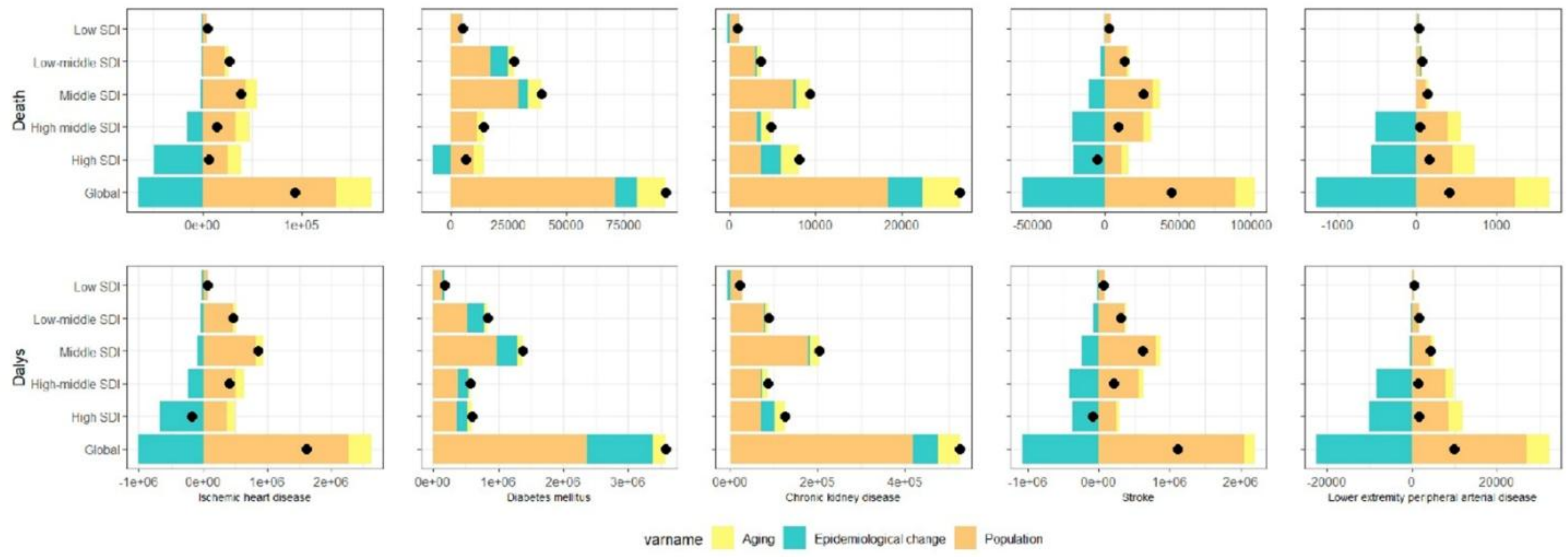

The figure's top row shows LPA-related Deaths, and the second row shows LPA-related DALYs. Each column represents a disease (eg, IHD, LEPAD). The y-axis indicates global and various SDI levels, while the x-axis shows the value range. Stacked bars are colour-coded for three factors: yellow for ageing, cyan for epidemiological change, and orange for population. SDI: socio-demographic index, DALYs: disability-adjusted life years, LPA: low physical activity, IHD: ischemic heart disease, LEPAD: lower extremity peripheral artery disease.

Table S1. Outline of JoGH guideline items

JoGH guideline item	Author's Response
1. Please list all papers published by each co-author in previous 3 years that were based on secondary analysis of a big data repository	**Hao Liu:** none published papers;<br>**Zhenhao Liu:**<br>**1.** Front Public Health. 2025 Jul 9; 13: 1576527. doi: 10.3389/fpubh.2025.1576527.<br>**2.** Front Public Health. 2025 May 29; 13: 1516365. doi: 10.3389/fpubh.2025.1516365.<br>**Yanqing Gong:** none published papers;<br>**Jingbin Guo:** none published papers;<br>**Xin Liu:** none published papers;<br>**Weiming Tang:**<br>**1.** JMIR Aging. 2025 Apr 30;8:e67437.<br>doi: 10.2196/67437.<br>**2.** BMC Med. 2024 Jun 18;22(1):250.<br>doi: 10.1186/s12916-024-03463-3.<br>**Weibin Cheng:**<br>**1.** JMIR Aging. 2025 Apr 30;8:e67437.<br>doi: 10.2196/67437.<br>**2.** Health Inf Sci Syst. 2025 Jan 27;13(1):19.<br>doi: 10.1007/s13755-025-00339-5.<br>**3.** BMC Med. 2024 Jun 18;22(1):250.<br>doi: 10.1186/s12916-024-03463-3.<br>**4.** Curr Probl Cardiol. 2023 Feb;48(2):101480.<br>doi: 10.1016/j.cpcardiol.2022.101480.<br>**Wen Jin:**<br>**1.** Curr Probl Cardiol. 2023 Feb;48(2):101480. doi: 10.1016/j.cpcardiol.2022.101480.
2. Please explain the key elements of your study design and the use of the available datasets that make your study an original scientific contribution	**1. Core innovations in study design**: Focusing on the association between low physical activity (LPA) and 5 chronic diseases, this study, for the first time, integrates multi-dimensional subgroup analyses (global, regional, national, SDI, and gender levels) with the APC (Age-Period-Cohort) and decomposition method to refine the patterns of disease burden disparities.<br>**2. Value of GBD database application**: By fully leveraging the GBD database's strengths of broad global coverage and complete time-series data, the study overcomes the limitations of single-region or single-disease studies. It systematically reveals the global and stratified characteristics of disease burden related to LPA for the 5 diseases, filling the gap in comprehensive multi-disease and multi-dimensional analyses in this field and providing scientific evidence for targeted interventions.
3. Please list all publications that addressed similar research questions in the same dataset and indicate where you cited them in your paper	**1. doi: 10.1681/ASN.0000000658:** In the "Introduction section", it is cited as "*Ischemic heart disease (IHD), stroke, lower extremity peripheral arterial disease (LEPAD), diabetes mellitus (DM), and chronic kidney disease (CKD) have become the main causes of death and disability globally [1,2].*"<br>**2. doi: 10.1016/S0140-6736(24)00933-4:** In the "Introduction section", it is cited as "*However, due to various reasons, many people don't meet the standards and are regarded as low physical activity (LPA) [15,16]*".<br>**3. doi：10.1016/S2214-109X(23)00355-8:** In the "Introduction section", it is cited as "*A study found that LPA is closely linked to the incidence and mortality of LEPAD, especially in low- and*

	middle-income countries and among the elderly [20]". **4. doi: 10.1136/bmj.i3857:** In the "Methods section", it is cited as "*The recognized threshold for physical inactivity is <600 MET minutes/week, yet it may not account for all increased death risks from insufficient physical activity [22]*".
4. Please explain how you addressed multiple testing through an appropriately rigorous statistical threshold and indicate this in the methods section	Thank you for your comment on multiple testing adjustment. We have supplemented relevant details in the revised Methods section to address potential type I errors, as follows: Based on the GBD database, our study included four multi-dimensional analyses: descriptive analyses (global/regional/national/SDI levels), sex-stratified analyses, Age-Period-Cohort (APC) analyses, and decomposition analyses (for independent factor effects). To control the family-wise error rate (FWER) at $\leqslant 0.05$, we applied the Bonferroni correction—a rigorous method for multiple testing. The corrected significance threshold (α_n) was calculated as $\alpha_n = 0.05/k$ (k=total number of independent tests, summed from comparisons/strata in each analysis). All inferential analyses (e.g., APC trend tests, decomposition factor significance tests) used this threshold to avoid false positives.
5. Please declare to what extent have AI chatbots been used in developing your paper and to which parts of the paper did they contribute	After the finalization of our manuscript, we utilized AI chatbots to polish the Introduction and Discussion sections. This was done to enhance the linguistic fluency of our manuscript and ensure it aligns with the submission requirements of the journal.

Table S2. Global Death and DALYs of Low Physical Activity caused by Lower extremity peripheral arterial disease (1990–2021).

Location	Death					DALYs				
	1990		2021		1990−2021	1990		2021		
	Number(95% UI)	ASMRs(95% UI)	Number (95% UI)	ASMRs (95% UI)	EAPCs(95% CI)	Number(95% UI)	ASDRs(95% UI)	Number(95% UI)	ASDRs(95% UI)	EAPCs(95% CI)
Global	1291 (375,2506)	0.17 (0.05, 0.33)	1697 (488, 3331)	0.08 (0.02, 0.15)	−2.78(−2.93, −2.63)	28225 (7801, 56466)	3.15 (0.88, 6.27)	38030 (10501, 76868)	1.68 (0.46, 3.39)	−2.38(−2.49, −2.27)
High SDI	6 83 (200, 1346)	0.23 (0.07, 0.46)	834 (242, 1667)	0.12 (0.03, 0.23)	−2.58(−2.81, −2.35)	13703 (3806, 27528)	4.51 (1.26, 9.06)	15405 (4375, 31371)	2.4(0.68, 4.89)	−2.38(−2.53, −2.23)
High−middle SDI	500 (142, 989)	0.25 (0.07, 0.5)	535 (152, 1073)	0.1 (0.03, 0.21)	−3.24(−3.39, −3.09)	10060 (2813, 20324)	4.38 (1.23, 8.78)	11553 (3141, 23722)	2.15 (0.59, 4.42)	−2.73(−2.87, −2.6)
Middle SDI	65(18, 129)	0.04 (0.01, 0.07)	198 (57, 400)	0.03 (0.01, 0.07)	−0.58(−0.69, −0.47)	2766 (693, 6011)	1.23 (0.31, 2.64)	7205 (1831, 15339)	1.06 (0.27, 2.26)	−0.7(−0.77, −0.62)
Low−middle SDI	26(7, 56)	0.03 (0.01, 0.05)	88(23, 185)	0.03 (0.01, 0.06)	0.76 (0.69, 0.82)	1189 (287, 2565)	0.9 (0.22, 1.93)	2792(701, 5962)	0.83 (0.21, 1.77)	−0.24(−0.29, −0.19)
Low SDI	14(3, 36)	0.04 (0.01, 0.09)	37(9, 93)	0.04 (0.01, 0.1)	0.46 (0.23, 0.69)	462 (107, 1079)	0.96 (0.22, 2.23)	1013(241, 2401)	0.92 (0.22, 2.17)	−0.14(−0.25, −0.03)
Andean Latin America	0(0, 1)	0.01(0, 0.02)	1(0, 3)	0.01 (0, 0.02)	1.25 (0.82, 1.68)	22(5, 51)	0.44 (0.1, 1.04)	60(14, 145)	0.39 (0.09, 0.93)	−0.38(−0.56, −0.2)
Australasia	30(8, 61)	0.5 (0.14, 1.03)	39(10, 83)	0.22 (0.06, 0.47)	−3.04(−3.28, −2.81)	499 (136, 1021)	7.94 (2.16, 16.28)	582(151, 1241)	3.49 (0.91, 7.43)	−2.91(−3.12, −2.7)
Caribbean	11(3, 23)	0.18 (0.05, 0.39)	25(7, 57)	0.16 (0.04, 0.37)	−0.25(−0.38, −0.12)	201(56, 428)	3.15 (0.88, 6.69)	441(115, 972)	2.93 (0.77, 6.46)	−0.03(−0.16, 0.09)
Central Asia	2(1, 4)	0.02(0, 0.04)	4(1, 8)	0.02 (0.01, 0.05)	1.34 (1.07, 1.62)	71(18, 157)	0.63 (0.16, 1.39)	106(27, 226)	0.57(0.15, 1.2)	−0.3(−0.45, −0.16)
Central Europe	98(28, 197)	0.29 (0.08, 0.58)	162 (46, 334)	0.25 (0.07,	−0.65(−0.86, −0.44)	1881 (522, 3800)	4.97 (1.39, 10.03)	2635(732, 5448)	4.14 (1.15, 8.57)	−0.86(−1.06, −0.66)

				0.51)						
Central Latin America	10(3, 21)	0.06 (0.02, 0.13)	18(5, 38)	0.03 (0.01, 0.06)	−2.96(−3.3, −2.63)	247(67, 516)	1.31 (0.36, 2.74)	470(126, 1014)	0.71 (0.19, 1.54)	−2.21(−2.45, −1.98)
Central Sub−Saharan Africa	5(1, 15)	0.14 (0.03, 0.41)	12(2, 36)	0.14 (0.03, 0.4)	−0.14(−0.42, 0.14)	134(27, 369)	2.88 (0.58, 7.88)	298(61, 835)	2.62 (0.55, 7.25)	−0.4(−0.65, −0.16)
East Asia	16(4, 34)	0.01(0, 0.02)	72(19, 158)	0.01 (0, 0.03)	0.61 (0.41, 0.81)	1921 (407, 4616)	1.01 (0.22, 2.38)	5623 (1249, 13105)	0.98 (0.22, 2.27)	−0.5(−0.65, −0.34)
Eastern Europe	284(79, 582)	0.44 (0.12, 0.91)	236 (63, 501)	0.24 (0.06, 0.5)	−2.62(−2.91, −2.32)	5086 (1405, 10601)	7.22 (2, 15.03)	4321 (1131, 9191)	4.3(1.13, 9.16)	−2.26(−2.53, −1.99)
Eastern Sub−Saharan Africa	5(1, 12)	0.04 (0.01, 0.1)	16(4, 39)	0.05 (0.01, 0.13)	1.24 (1.07,1.4)	129(31, 304)	0.82 (0.19, 1.92)	390(100, 883)	1.08 (0.28, 2.45)	0.89 (0.77,1.02)
High−income Asia Pacific	28(7, 56)	0.06 (0.02, 0.13)	114 (33, 230)	0.06 (0.02, 0.11)	0.04(−0.31, 0.4)	1117 (281, 2454)	2.19 (0.55, 4.78)	2416(627, 5049)	1.5(0.38, 3.17)	−1.2(−1.32, −1.09)
High−income North America	273(78, 560)	0.27 (0.08, 0.56)	284 (83, 589)	0.14 (0.04, 0.29)	−3.15(−3.53, −2.76)	5259 (1423, 10941)	5.23 (1.41, 10.9)	5627 (1606, 11722)	2.97 (0.85, 6.18)	−2.75(−3.07, −2.43)
North Africa and Middle East	8(2, 19)	0.02 (0.01, 0.05)	21(5, 50)	0.02 (0.01, 0.06)	0.21(−0.02, 0.44)	375(90, 850)	0.95 (0.23, 2.15)	794(193, 1826)	0.73 (0.17, 1.69)	−0.95(−1.07, −0.83)
Oceania	0(0, 0)	0.01(0, 0.02)	0(0, 0)	0.01 (0, 0.02)	0.03(−0.38, 0.44)	5(1, 11)	0.73 (0.17, 1.67)	10(3, 24)	0.61 (0.15, 1.44)	−0.56(−0.62, −0.51)
South Asia	14(3, 34)	0.01(0, 0.03)	56(13, 135)	0.02 (0, 0.05)	1.56 (1.35, 1.77)	873 (202, 2037)	0.71 (0.16, 1.64)	2158(503, 5055)	0.62 (0.15, 1.46)	−0.2(−0.31, −0.09)
Southeast Asia	3(1, 7)	0.01(0, 0.01)	11(3, 24)	0.01 (0, 0.02)	0.17 (0.05, 0.29)	485 (107, 1154)	0.82 (0.18, 1.95)	999(233, 2323)	0.6(0.14, 1.38)	−1.08(−1.11, −1.05)
Southern Latin America	6(2, 13)	0.06 (0.02, 0.12)	10(2, 22)	0.04 (0.01, 0.09)	−0.93(−1.48, −0.37)	201(51, 457)	1.69 (0.44, 3.83)	262(61, 624)	1.06 (0.25, 2.52)	−1.26(−1.55, −0.97)
Southern Sub−Saharan Africa	6(2, 14)	0.1 (0.03, 0.22)	16(4, 33)	0.12 (0.03, 0.25)	0.49 (0.32, 0.65)	181(47, 381)	2.61 (0.68, 5.51)	419(112, 872)	2.77 (0.74, 5.78)	0.14(0,0.28)
Tropical Latin America	32(8, 66)	0.18 (0.05, 0.37)	107 (29, 225)	0.16 (0.04, 0.34)	−0.65(−0.84, −0.47)	719 (185, 1534)	3.43 (0.89, 7.3)	2191(583, 4667)	3.18 (0.85, 6.78)	−0.59(−0.76, −0.42)
Western Europe	449 (129, 891)	0.28 (0.08, 0.56)	464 (133, 920)	0.13 (0.04, 0.26)	−2.3(−2.56, −2.03)	8556 (2349, 17160)	5.22 (1.44, 10.47)	7602 (2121, 15418)	2.5(0.69, 5.08)	−2.35(−2.53, −2.17)
Western Sub−Saharan Africa	11(2, 29)	0.07 (0.02, 0.19)	28(6, 68)	0.08 (0.02, 0.2)	0.2(0.1, 0.31)	265(63, 627)	1.4 (0.33, 3.33)	626(150, 1455)	1.48 (0.35, 3.47)	0.07(−0.02, 0.16)

Table S3. Global Death and DALYs of Low Physical Activity caused by Ischemic heart disease (1990–2021).

Location	Death					DALYs				
	1990		2021		1990−2021	1990		2021		1990−2021
	Number(95% UI)	ASMRs(95% UI)	Number(95% UI)	ASMRs(95% UI)	EAPCs(95% CI)	Number(95% UI)	ASDRs(95% UI)	Number(95% UI)	ASDRs(95% UI)	EAPCs(95% CI)
Global	123863(52950, 201328)	14.82 (6.27, 24.26)	229586 (100303, 376343)	10.41 (4.54, 17.07)	−1.19(−1.24, −1.15)	2149825 (932677, 3454358)	228.86 (98.4, 369.97)	3758534 (1668371, 6122182)	164.37 (72.81, 268.1)	−1.16(−1.22, −1.11)
High SDI	44700 (18091, 77821)	14.94 (6.04, 26.08)	35724 (14157, 60785)	5.05 (2.02, 8.57)	−3.78(−3.92, −3.64)	673755 (274080, 1159158)	221.1 (90.05, 381.07)	490882 (198608, 834878)	76.44 (31.27, 129.62)	−3.72(−3.92, −3.53)
High−middle SDI	34301 (14223, 57092)	17.09(7.1, 28.43)	66449 (28092, 115576)	12.81 (5.43, 22.3)	−1.04(−1.18, −0.89)	544861 (225340, 905648)	240.28 (99.5, 399.22)	947844 (396808, 1643142)	178.16 (74.7, 309.04)	−1.12(−1.26, −0.97)
Middle SDI	25157 (10232, 42179)	13.06 (5.28, 21.82)	78311 (33360, 133217)	12.86 (5.45, 21.97)	0.05(−0.03, 0.12)	503492 (207006, 846835)	215.8 (88.14, 362.03)	1359707 (588830, 2291671)	202.86 (87.28, 343.43)	−0.2(−0.23, −0.16)
Low−middle	16398	12.95 (5.35,	42150	13.31 (5.5,	0.22 (0.13,	356945	240.24	821724	231.73	−0.1(−0.1

SDI	(6840, 27374)	21.64)	(17648, 71765)	22.62)	0.31)	(150591, 595671)	(100.56, 401.08)	(351647, 1394152)	(98.02, 393.28)	5, −0.05)
Low SDI	3109 (1267, 5386)	7.19(2.87, 12.46)	6711 (2743, 11699)	6.89(2.77, 12)	0.08(−0.12 , 0.29)	67504 (27887, 116939)	128.71(52.3 8, 223)	134566 (56228, 233421)	116.65 (47.89, 202.77)	−0.24(−0. 38, −0.1)
Andean Latin America	174(57, 369)	3.79(1.24, 8.08)	351(113, 760)	2.33 (0.75, 5.04)	−1.87(−2. 23, −1.5)	2873(937, 6117)	58.45(19.06, 124.47)	5591(1818, 12138)	36.22 (11.76, 78.58)	−1.83(−2. 21, −1.46)
Australasia	1175(404, 2314)	19.5(6.7, 38.3)	914(330, 1812)	5.17 (1.86, 10.27)	−4.54(−4. 64, −4.45)	18284(6183, 37121)	290.97 (98.39, 590.26)	11976(4275, 23975)	73.18 (26.01, 147.59)	−4.71(−4. 87, −4.56)
Caribbean	840(321, 1568)	13.83 (5.31, 25.77)	1146(429, 2187)	7.59 (2.85, 14.47)	−2.02(−2. 21, −1.83)	14486(5567, 26997)	221.08 (85.13, 411.58)	19534(7405, 37111)	130.9 (49.7, 248.53)	−1.77(−1. 95, −1.58)
Central Asia	1979(776, 3475)	19.19(7.5, 33.72)	2470(972, 4460)	15.49 (6.12, 27.82)	−1.04(−1. 27, −0.82)	30359 (11899, 53409)	274.53 (107.25, 483.16)	37809 (14883, 69040)	216.68 (85.54, 392.91)	−1.22(−1. 49, −0.95)
Central Europe	7809 (3216, 13344)	23.06 (9.48, 39.42)	8889 (3754, 15047)	13.31 (5.62, 22.56)	−2.03(−2. 12, −1.94)	123247 (51044, 211608)	333.19 (137.59, 571.78)	119966 (50365, 203638)	182.52 (76.62, 310.42)	−2.25(−2. 34, −2.15)
Central Latin America	1072(367, 2031)	6.03(2.06, 11.37)	3060(1157, 5781)	4.8 (1.82, 9.07)	−0.87(−1. 3, −0.44)	19660(6877, 37507)	98.78 (34.25, 187.69)	51390 (19344, 96886)	77.8 (29.29, 146.72)	−0.93(−1. 37, −0.49)
Central Sub−Saharan Africa	284(94, 631)	7.66(2.52, 16.5)	627(203, 1357)	7(2.29, 15.07)	−0.51(−0. 6, −0.41)	6057(1991, 13607)	130.03 (42.84, 285.29)	12627(4043, 27544)	115.04 (37.34, 248.55)	−0.62(−0. 73, −0.51)
East Asia	13012 (4241, 27102)	10.23(3.5, 20.91)	58521 (19452, 116967)	12.46 (4.18, 24.79)	1.07 (0.74, 1.4)	234451 (73477, 495008)	143.89 (47.49, 298.34)	833473 (267962, 1691440)	162.01 (52.9, 326.33)	0.77 (0.5, 1.04)
Eastern Europe	15616 (6018, 29113)	25.88 (10.02, 47.96)	20020 (7540, 36663)	20.04 (7.53, 36.77)	−1.15(−1. 56, −0.73)	232812 (87908, 441961)	350.03 (133.06, 659.1)	274799 (101130, 514133)	274.47 (100.75, 514.87)	−1.15(−1. 57, −0.74)
Eastern Sub−Saharan Africa	230(87, 435)	1.54(0.59, 2.89)	550(212, 1021)	1.65 (0.62, 3.09)	0.12 (0.08, 0.17)	5295(1999, 10110)	29.48 (11.19, 55.74)	11996(4754, 22025)	30.25 (11.71, 55.93)	−0.02(−0. 06, 0.02)
High−income Asia Pacific	2642(785, 5778)	5.66(1.66, 12.42)	3591(1076, 7596)	2.04 (0.61, 4.36)	−3.23(−3. 41, −3.06)	45101 (13736, 97969)	88.46 (26.69, 192.55)	49601 (14956, 106599)	34.69 (10.42, 75.93)	−2.99(−3. 12, −2.86)
High−income North America	12296 (3417, 28200)	12.14 (3.37, 27.89)	10953 (3412, 22803)	5.34 (1.67, 11.14)	−3.03(−3. 26, −2.8)	171418 (46631, 397973)	170.31 (46.28, 396.79)	147869 (45931, 311626)	75.9 (23.62, 160.65)	−3(−3.31, −2.68)
North Africa and Middle East	11634 (4885, 19057)	33.51 (13.87, 55.23)	24110 (10306, 39687)	25.11 (10.63, 41.52)	−0.96(−1. 04, −0.88)	252016 (107778, 409845)	611.14 (257.96, 999.5)	485834 (211823, 792620)	434.55 (187.37, 712.39)	−1.21(−1. 27, −1.14)
Oceania	71(27, 136)	11.17(4.3, 21.06)	165(60, 323)	9.64 (3.47, 18.78)	−0.51(−0. 56, −0.46)	1939(746, 3748)	243.2 (93.38, 465.18)	4431(1648, 8730)	211.21 (77.29, 414.11)	−0.49(−0. 56, −0.43)
South Asia	15522 (5630, 29524)	13.22 (4.87, 24.83)	48403 (18508, 89831)	15.16 (5.78, 27.86)	0.58 (0.41, 0.76)	343375 (122137, 664156)	247.88 (89.68, 473.02)	916724 (351617, 1727711)	255.27 (97.67, 476.59)	0.08(−0.04 , 0.2)
Southeast Asia	6185 (2409, 11174)	11.81 (4.61, 21.24)	19518 (7655, 35234)	13.47 (5.35, 23.97)	0.39 (0.26, 0.52)	128749 (49854, 235171)	212.07 (82.41, 384.68)	387322(14881 2, 714637)	237.44 (92.45, 431.81)	0.33 (0.24, 0.42)
Southern Latin America	542(174, 1188)	5.09(1.63, 11.15)	470(148, 1016)	1.86 (0.59, 4.02)	−2.81(−3. 05, −2.58)	8597(2778, 18858)	74.65 (24.06, 163.71)	6854(2220, 14712)	27.76 (9.03, 59.52)	−2.86(−3. 11, −2.62)
Southern Sub−Saharan Africa	615(221, 1184)	10.71 (3.88, 20.43)	1397(513, 2615)	12.24 (4.54, 22.73)	0.23(−0.09 , 0.55)	12129(4302, 23826)	184.78 (66.18, 359.33)	27007(9760, 51482)	200.61 (73.27, 378.34)	0.05(−0.26 , 0.36)
Tropical Latin America	2886(951, 5904)	15.74 (5.36, 31.55)	4869(1487, 9924)	7.3 (2.24, 14.78)	−2.3(−2.4 9, −2.12)	53027 (16614, 112358)	251.98 (81.57, 522.42)	87411 (25691, 185597)	127.22 (37.66, 268.23)	−2.1(−2.2 9, −1.9)
Western Europe	28246 (11692, 48400)	17.2(7.12, 29.56)	17421 (7025, 29709)	5.08 (2.05, 8.69)	−4.2(−4.3 2, −4.07)	425279 (175476, 727186)	258.07(106. 56, 442.56)	223511 (90847, 382920)	72.61 (29.54, 124.87)	−4.38(−4. 52, −4.25)
Western Sub−Saharan Africa	1034(384, 2067)	5.9(2.17, 11.94)	2141(782, 4025)	5.75 (2.09, 10.77)	−0.08(−0. 18, 0.01)	20671(7762, 40983)	100.78(37.5 8, 201.35)	42811 (15798,80688)	96.31 (35.32, 180.82)	−0.15(−0. 25, −0.05)

Table S4. Global Death and DALYs of Low Physical Activity caused by Stroke (1990–2021).

Location	Death					DALYs				
	1990		2021		1990−2021	1990		2021		1990−202 1
	Number(95% UI)	ASMRs(95 % UI)	Number(95% UI)	ASMRs(9 5% UI)	EAPCs(95 % CI)	Number(95% UI)	ASDRs(95 % UI)	Number(95% UI)	ASDRs(95 % UI)	EAPCs(95 % CI)

Global	90346(−31089, 209511)	9.5(−5.69, 24.28)	135766(−65733, 337963)	5.9(−3.38, 15.17)	−1.77(−1.88, −1.67)	2059855(35568, 4082172)	198.62(−27.49, 422.35)	3172145(−148487, 6581638)	133.79(−13.86, 284.8)	−1.52(−1.61, −1.43)
High SDI	20930(−15992, 57112)	6.82(−5.61, 18.96)	15178(−17396, 47072)	2.27(−2.12, 6.57)	−3.9(−4.06, −3.73)	430620(−117343, 978128)	140.47(−39.52, 319.63)	346496(−147029, 838490)	59.38(−13.73, 132.79)	−3.1(−3.27, −2.92)
High−middle SDI	32226(−13168, 77503)	13.52(−9.41, 36.01)	41203(−27676, 111277)	7.63(−5.62, 21.09)	−2.24(−2.45, −2.03)	701099(−28103, 1441530)	267.96 (−57.67,593.52)	907191(−160088, 2025712)	164.51(−35.65, 373.88)	−1.97(−2.15, −1.79)
Middle SDI	22336(−2677, 47194)	9.54(−4.77, 23.24)	48706(−16961, 115859)	7.2(−3.99, 18.49)	−1.02(−1.1, −0.94)	555363 (82330, 1039954)	208.49 (−15.55,430.4)	1171127 (22975, 2379458)	160.45(−17.78, 345.99)	−0.98(−1.04, −0.92)
Low−middle SDI	11667(−579, 24376)	8.38(−3.01, 19.83)	24881(−4214, 54323)	7.12(−2.91, 17.12)	−0.58(−0.64, −0.52)	291413 (55789, 539005)	185.15 (1.91,373.18)	600883 (73905, 1140987)	157.47(−3.23, 319.96)	−0.63(−0.69, −0.57)
Low SDI	3047(82, 6370)	6(−2.12, 14.57)	5655(−500, 12382)	4.97(−2.08, 12.46)	−0.69(−0.75, −0.63)	78375 (17887, 147792)	134.49 (2.03,279.67)	143352 (24016, 276098)	110.32(−3.36, 233.94)	−0.78(−0.85, −0.72)
Andean Latin America	156(−67, 399)	3.14(−1.79, 8.46)	258(−179, 693)	1.67(−1.26, 4.56)	−2.31(−2.55, −2.08)	3535(−163, 7752)	66.89 (−9.58,152.79)	5832(−1148, 13270)	36.75(−9.04, 84.99)	−2.18(−2.42, −1.95)
Australasia	389(−319, 1121)	6.25(−6.17, 18.93)	297(−482, 1073)	1.69(−2.53, 5.91)	−4.45(−4.59, −4.31)	7841(−2541, 19023)	121.33 (−50.64,304.35)	6460(−4427, 17643)	42.26(−21.3, 108.32)	−3.6(−3.8, −3.4)
Caribbean	474(−192, 1131)	7.18(−4.21, 18.28)	743(−339, 1888)	4.98(−2.21, 12.59)	−1.14(−1.21, −1.07)	10306(−303, 21075)	148.58 (−18.47,316.15)	16118(−734, 34405)	108.55(−4.68, 231.38)	−0.96(−1.04, −0.89)
Central Asia	988(−334, 2322)	8.58(−3.97, 21.13)	1264(−531, 3091)	6.87(−4.2, 18)	−1.19(−1.47, −0.9)	24058 (883, 48520)	194.64 (−8.57,406.65)	31488(301, 65063)	150.29(−21.11, 332.42)	−1.32(−1.62, −1.02)
Central Europe	6175(−3322, 15282)	16.43(−12.3, 43.37)	4898(−4100, 13941)	7.42(−5.96, 20.89)	−2.93(−3.08, −2.78)	125669(−18377, 269573)	313.79 (−85.28,704.72)	94995(−31832, 224557)	149.27(−42.46, 345.8)	−2.76(−2.89, −2.62)
Central Latin America	726(−307, 1795)	3.75(−2.44, 10.03)	1200(−745, 3184)	1.83(−1.31, 5.01)	−2.53(−2.8, −2.25)	17066(−252, 35776)	80.09 (−12.97,178.73)	28481(−3097, 61783)	41.89(−7.48, 93.56)	−2.34(−2.64, −2.03)
Central Sub−Saharan Africa	340(9, 762)	7.43(−3.53, 19.74)	694(−100, 1684)	6.66(−3.65, 18.67)	−0.52(−0.58, −0.47)	8903 (1701, 18520)	163.13 (−18.4,380.73)	17862(1792, 38830)	142.13(−23.94, 344.42)	−0.61(−0.67, −0.55)
East Asia	20090(−2950, 44727)	10.34(−6.88, 27.03)	46112(−22355, 123213)	8.16(−5.8, 23.57)	−0.78(−0.98, −0.58)	496641 (45002, 1016840)	221.42 (−45.55,501.88)	1078776(−101724, 2466468)	177.52(−41.85, 430.51)	−0.74(−0.87, −0.61)
Eastern Europe	12945(−6500, 32279)	18.39(−13.5, 49.25)	9908(−8216, 28740)	10.06(−7.87, 28.88)	−2.76(−3.24, −2.27)	268889(−32709, 586710)	356.11 (−91.45,814.73)	198101(−61175, 481481)	200.91(−55.54, 484.67)	−2.65(−3.1,− 2.19)
Eastern Sub−Saharan Africa	544(−14, 1193)	3.33(−1.47, 8.47)	1062(−118, 2391)	2.91(−1.26, 7.42)	−0.54(−0.58, −0.5)	14839 (2824, 29343)	78.07 (−4.06,170.5)	29016(4530, 57130)	68.74(−3.26, 148.46)	−0.52(−0.56, −0.48)
High−income Asia Pacific	3836(−1997, 10089)	7.62(−5.51, 21.64)	3006(−3926, 10154)	1.79(−1.61, 5.36)	−5.08(−5.25, −4.91)	92942(−5446, 200815)	171 (−30.05,390)	70972(−36576, 185244)	57.15(−8.44, 128.93)	−3.91(−4.06, −3.75)
High−income North America	3221(−3028, 10031)	3.15(−3.03, 9.86)	3613(−4492, 11691)	1.78(−2.08, 5.67)	−2.37(−2.72, −2.01)	68286(−28397, 178720)	68.7 (−27.25,178.49)	86753(−40385, 222568)	46.89(−17.67, 116.81)	−1.65(−1.94, −1.35)
North Africa and Middle East	7356(−429, 15438)	18.52(−6.99, 44.05)	13553(−2092, 29356)	12.42(−5.25, 29.94)	−1.33(−1.38, −1.29)	187838 (38703, 346735)	417.29 (9.4,840.45)	350763 (60523, 651357)	284.11 (2.37, 571.76)	−1.32(−1.36, −1.28)
Oceania	39(4, 85)	6.11(−2.31, 15.55)	80(−2, 180)	4.85(−2.12, 12.59)	−0.91(−0.98, −0.84)	1214(332, 2367)	155.16 (3.8,335.39)	2488(573, 4906)	124.23(−0.86, 270.37)	−0.87(−0.93, −0.8)
South Asia	7935(−161, 17391)	6.24(−2.07, 15.34)	18174(−3878, 42584)	5.14(−2.48, 13.25)	−0.86(−0.97, −0.76)	195260 (35037, 390298)	135.31 (−0.09,290.94)	414606 (30286, 862552)	107.23(−9.68, 238.16)	−1.04(−1.16, −0.92)
Southeast Asia	5877(−798, 12698)	10.23(−4.3, 24.81)	16523(−3246, 37478)	10.5(−4.57, 26.14)	0.09(−0.07, 0.24)	155528 (23429, 293273)	238.01 (−4.69,487.41)	425823 (47929, 833628)	242.64(−9.55, 510.08)	0.05(−0.06, 0.16)
Southern Latin America	562(−325, 1512)	4.77(−3.66, 13.64)	430(−390, 1277)	1.71(−1.5, 5.04)	−2.98(−3.13, −2.83)	12237(−1894, 28321)	98.25 (−26.25,237.17)	9618(−3086, 23533)	39.5(−11.27, 95.32)	−2.76(−2.9, −2.62)
Southern Sub−Saharan Africa	480(−181, 1170)	7.63(−4.91, 20.4)	1257(−414, 2983)	9.69(−6.37, 25.94)	0.82(0.33, 1.31)	13139 (451, 26845)	187.36 (−24.77,411.72)	31385(919, 63428)	213.05(−39.2, 472.98)	0.42 (0.02, 0.83)
Tropical Latin America	2825(−833, 6605)	13.31(−8.06, 34.61)	3362(−1732, 8773)	4.94(−2.89, 13.2)	−3.1(−3.22, −2.99)	63667 (1435, 132084)	270.42 (−45.9,604.53)	73191(−7828, 163089)	104.6(−16.68, 238.1)	−3.09(−3.23, −2.94)
Western Europe	13832(−13051, 39808)	8.32(−8.11, 24.14)	6477(−10142, 22209)	1.97(−2.56, 6.29)	−4.86(−5.02, −4.7)	252116(−115813, 613646)	153.12 (−66.56,368.58)	123711(−102424, 341334)	44.85(−22.75, 110.86)	−4.17(−4.36, −3.97)

Western Sub−Saharan Africa	1556(−216, 3580)	7.6(−3.67, 19.71)	2855(−373, 6405)	6.58(−2.99, 16.72)	−0.49(−0.57, −0.42)	39884 (5237, 81318)	173.42 (−11.59,383.02)	75707 (12063, 147920)	149.85(−6.07, 321.16)	−0.51(−0.58, −0.44)
